# TRAF6, a gga-miR-7b Target, Promotes *Eimeria tenella*-Induced Inflammation and Apoptosis in Chickens by Activating NF-κB Pathway

**DOI:** 10.3390/biom16050655

**Published:** 2026-04-28

**Authors:** Jianqiang Tang, Jin Zhang, Meihui Tang, Liyue Dong, Areej Arif, Genxi Zhang, Tao Zhang, Kaizhou Xie, Guojun Dai

**Affiliations:** 1 School of Veterinary Medicine, Yangzhou University, Yangzhou 225009, China; 2 College of Animal Science and Technology, Yangzhou University, Yangzhou 225000, China; 3 College of Animal Science and Technology, Henan Agricultural University, Zhengzhou 450002, China

**Keywords:** chicken, *Eimeria tenella*, TRAF6, immune response, miRNA

## Abstract

Chicken coccidiosis, a severe intestinal parasitic disease caused by *Eimeria* protozoa, causes substantial annual economic losses to the global poultry industry. This study focused on investigating the role of tumor necrosis factor receptor (TNFR)-associated factor 6 (TRAF6) in modulating chicken innate immune responses against *Eimeria tenella* (*E. tenella*) infections. Here, we show that *TRAF6* is sensitive to the process of *E. tenella* infection, and cecal tissue responds to the early infection of *E. tenella* by up-regulating the expression of *TRAF6*. Specifically, *TRAF6* overexpression enhances *E. tenella*-induced activation of the NF-κB pathway (a core innate immune signaling cascade), thereby promoting host inflammatory cytokines production and cell apoptosis, while *TRAF6* knockdown mitigates these pathological effects. Mechanistically, *TRAF6*-mediated regulation of NF-κB pathway activation and inflammatory responses during *E. tenella* infection can be specifically targeted by key microRNAs (miRNA), gga-miR-7b, in chickens. Taken together, this study identifies that TRAF6 plays an important regulatory role in innate immune response against *E. tenella* infection, providing novel insights into host–parasite interactions and potential targets for coccidiosis control.

## 1. Introduction

Chicken coccidiosis is an intestinal parasitic disease caused by protozoa of the *Eimeria* genus, among which *Eimeria tenella* (*E. tenella*) is regarded as one of the most pathogenic species [1,2,3]. It primarily parasitizes the cecum and is infectious to chickens of all ages, with particularly severe harm to chicks [4,5]. In chickens, coccidial infection affects intestinal mucosal ulceration, bloody diarrhea, and hemorrhagic lesions of the intestinal wall, accompanied by systemic symptoms such as lethargy, reduced feed intake, inflammation, suppression of the immune system, and disorders of nutrient metabolism [6,7,8]. These deleterious effects seriously jeopardize poultry productivity and welfare, causing substantial economic losses to the poultry industry. Currently, the prevention and control of coccidiosis mainly relies on the use of chemically synthesized anticoccidial drugs and coccidial vaccines [9,10]. However, the long-term use of anticoccidial drugs has led to drug resistance and residues in chicken, and public food safety hazards [9,11]. Moreover, the issues related to the safety, costs, and application of coccidial vaccines also have severely restricted the effectiveness of prevention and control [12]. Given the limitations of conventional anticoccidial drugs and vaccines, alternative control strategies increasingly focus not only on directly targeting the parasite, but also on modulating host defense pathways that influence disease severity and tissue damage. In avian coccidiosis, a clearer understanding of hosts’ innate immune signaling is important for the rational development of non-antibiotic interventions, the improvement of vaccine efficacy, and the identification of host immune markers associated with disease resistance.

Tumor necrosis factor receptor (TNFR)-associated factor 6 (TRAF6), a member of the tumor necrosis factor receptor-associated factor (TRAF) family, is an adaptor protein that broadly mediates protein signal transduction through its TRAF domain and a RING finger domain that possesses non-conventional E3 ubiquitin ligase activity [13]. TRAF6 functions as a key regulatory molecule in the activation of the NF-κB signaling pathway mediated by the interleukin-1 receptor (IL-1R) [14]. Additionally, it serves as a critical downstream molecule for multiple immune-regulatory receptors, including the members of the TNFR superfamily, toll-like receptors (TLRs), and T cell receptors (TCRs) [15,16]. In the immune system, TRAF6-mediated signaling is essential for the development, homeostasis, and activation of B cells, T cells, and myeloid immune cells (including macrophages, dendritic cells, and osteoclasts) [17,18,19,20]. Recent studies have shown that TRAF6 plays a significant regulatory role in host immune responses against pathogenic infections. Deng et al. [21]. found that the VP3 protein of *Avibirnavirus* can block nuclear factor kappaB (NF-κB) activation via inducing the autophagic degradation of TRAF6, thereby evading the host innate immune response during viral infection. Wu et al. [22]. revealed that TRAF6 can be involved in the regulation of macrophage inflammatory responses during polymicrobial sepsis. In addition to viral and bacterial settings, TRAF6-dependent signaling has also been implicated in the immune response to the apicomplexan protozoan *Toxoplasma gondii* [20]. Importantly, although *E. tenella* is a eukaryotic protozoan parasite, previous studies in chickens have shown that *E. tenella* infection or sporozoite stimulation can activate innate immune receptors and signaling molecules upstream of TRAF6, including TLR4, TLR15, MyD88, and NF-κB [23,24]. Therefore, it is biologically plausible that TRAF6 participates in the host inflammatory response to *E. tenella* infection through TLR-related innate immune signaling.

However, to date, little is known about the expression patterns, functions and regulation of *TRAF6* in chicken during *E. tenella* infection. In the study, we identified the characteristics of chicken TRAF6, examined the morphological structure of cecal tissues and the expression levels of inflammatory cytokines and *TRAF6*, and further investigated the correlations between *TRAF6* and immune-related genes in the cecal tissues at different stages after *E. tenella* infection. Then, in vitro gain and loss-of-function assays in DF-1 cells were conducted to explore the functions and regulatory mechanism of *TRAF6* on the host innate immune response against *E. tenella* infections. The findings of this study expand new research fields for *TRAF6* molecules and provide in-depth references for identifying *TRAF6* as a key regulatory molecule of inflammatory response during chicken coccidial infection.

## 2. Materials and Methods

### 2.1. Experimental Animals and Oocysts

**Figure 1:**
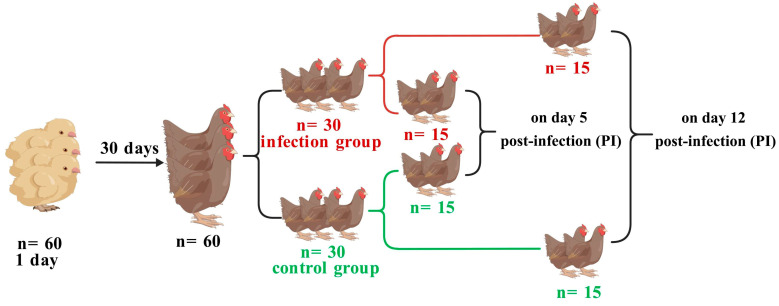
Schematic of experimental design.

A total of sixty 1-day-old female Jinghai Yellow chickens were randomly selected from Jiangsu Jinghai Poultry Industry Group Co., Ltd. (Nantong, China). Each chicken was separately raised in a pathogen-free animal house, with free access to feed and water (without any anticoccidial drugs), and not vaccinated against coccidia. At 30 days of age, chicken feces were randomly selected, placed in saturated saline, and observed under a microscope for the presence of coccidian oocysts in the supernatant to ensure no coccidial infection. The 60 female chickens were randomly divided into two groups: a control group and infection group, with 30 birds in each group. The infection group was orally gavaged with 3.5 × 10^4^ sporulated *E. tenella* oocysts per chicken, while the control group received an equal volume of normal saline. Five days post-infection (120 h; dpi) (the early stage), 15 chickens were randomly selected from both the control and infection groups to perform sample collection, respectively. Meanwhile, the remaining chickens in each group continued to be raised until 12 dpi (288 h; the late stage). The experimental procedure is referred to in Figure 1. *E. tenella* oocysts (YZ strain) were gifted by the Parasitology Department of the College of Veterinary Medicine, Yangzhou University [2].

### 2.2. Sample Collection

At 5 and 12 dpi, chickens from each group (a total of 15 birds per group) were anesthetized with Zoletil 50 (12–15 mg/kg, IM) prior to blood collection by cardiac puncture. Adequate anesthetic depth was confirmed by the loss of palpebral reflex and the absence of response to toe pinch. Blood samples were placed into heparin sodium anticoagulant tubes. The tubes were immediately centrifuged at 4000× *g* for 5 min at 4 °C, and the supernatant plasma was harvested and stored at −20 °C. Subsequently, the chickens of each group were euthanized by rapid cervical dislocation [25]. The cecal tissues were aseptically collected and placed into cryotubes containing RNA preservation solution. The samples were stored at −20 °C for subsequent experiments.

### 2.3. Intestinal Morphology

On 5 and 12 dpi, approximately 2 cm intestinal segments from the anterior 1/4 of the cecal tissue were collected from chickens in both the control and infection groups, respectively. The samples were fixed in 4% paraformaldehyde for 24 h, dehydrated with gradient ethanol, clarified with xylene, embedded in paraffin, and sectioned at 5 μm thickness. Then the sections were deparaffinized and rehydrated, and stained with hematoxylin-eosin (H&E) (Solarbio, Beijing, China) according to the manufacturer’s instructions. The slides were observed under a light microscope and photographed (Nikon Corporation, Tokyo, Japan) [26]. “100×” represent magnifications of 100 times.

### 2.4. DF-1 Cell Culture and Transfection

Chicken DF-1 cells (a chicken embryo fibroblast cell line) were provided by the Laboratory of Guobin Chang, College of Animal Science and Technology, Yangzhou University, and maintained in complete DMEM (Gibco, Grand Island, NY, USA), supplemented with 10% fetal bovine serum (FBS) (Gibco, Grand Island, NY, USA) and 1% penicillin-streptomycin solution (Solarbio, Beijing, China). Fresh complete medium was prepared prior to each use, and cells were cultured in a humidified incubator at 37 °C with 5% CO_2_. The cell transfection was performed using jetPRIME^®^ transfection reagent (Polyplus, Illkirch, France) following the manufacturer’s protocol. Cell viability after transfection was evaluated by CCK-8 assay, and the corresponding data are provided in Supplementary Figure S1. The transfection concentrations were as follows: pcDNA3.1-TRAF6 and pcDNA3.1 vectors were 800 ng/μL; siRNA-TRAF6 and siRNA-NC into cells were 20 μmol/L; gga-miR-7b mimic and mimic-NC were 50 nmol/L, gga-miR-7b inhibitor, inhibitor-NC were 100 nmol/L.

### 2.5. RNA Oligonucleotides and Plasmid Construction

For the *TRAF6* overexpression, the coding sequence (CDS) region of the *TRAF6* gene (XM_004941548.5, NCBI) was amplified and cloned into the multiple cloning site (MCS) of the empty pcDNA3.1(+) vector between the restriction enzyme sites *BamHI* (GGATCC) and *EcoRI* (GAATTC), thus constructing the *TRAF6* gene overexpression vector (pcDNA3.1-TRAF6). Additionally, four siRNA targeting the *TRAF6* gene, a negative control siRNA (siRNA-NC), and FAM-labeled siRNA oligonucleotides were designed, and synthesized by Gene Pharma (Suzhou, China) (Table S1). Gga-miR-7b mimic, mimic-NC (negative control) and gga-miR-7b inhibitor, inhibitor-NC were designed and synthesized by Ribobio (Guangzhou, China) (Table S2). The sequences of all oligonucleotides and the *TRAF6* CDS are provided in Supplementary Materials.

### 2.6. Effect of TRAF6 on the Inflammatory Response of DF-1 Cells Infected with Sporozoites

Chicken DF-1 cells were uniformly seeded into 12-well plates at 3 × 10^5^ cells/well, and cultured in a constant temperature incubator at 37 °C with 5% CO_2_. When the cell reached 70% confluence, pcDNA3.1-TRAF6, pcDNA3.1, or siRNA-TRAF6, and siRNA-NC were transfected into host cells in vitro, respectively. After 24 h transfection, purified *E. tenella* sporozoites were added at an MOI of 3:1 (sporozoites: cells; approximately 9 × 10^5^ sporozoites per well), and incubated for an additional 6 h before sample collection, as described in our previous study [27]. Subsequently, cell supernatants and cell samples were collected to detect the expression levels of inflammatory cytokines by enzyme-linked immunosorbent assays (ELISA) and real-time quantitative PCR (RT-qPCR), respectively.

### 2.7. The Effect of gga-miR-7b on TRAF6 Expression

Chicken DF-1 cells were uniformly seeded into a 12-well plate until reaching 70% confluence. The gga-miR-7b mimic, mimic-NC or gga-miR-7b inhibitor, and inhibitor NC were transfected into cells, respectively. Each treatment group had three biological replicates. After transfection for different time periods (12, 24, 36, and 48 h), cell samples were collected, and *TRAF6* gene mRNA expression level was detected by RT-qPCR.

### 2.8. RNA Isolation, Reverse Transcription and Real-Time Quantitative PCR

Total RNA was extracted from tissues or chicken DF-1 cells using TRIZOL reagent (Tiangen, Beijing, China) following the manufacturer’s protocol. RNA purity and optical density (OD) were measured with a Nanodrop-2000 spectrophotometer (Thermo Fisher Scientific, Waltham, MA, USA). To reduce potential interference from residual genomic DNA or plasmid DNA, RNA samples were treated with the gDNA removal reagent included in the HiScript III RT SuperMix kit before cDNA synthesis. For plasmid-mediated *TRAF6* overexpression analysis, a no-reverse-transcription (no-RT) control was included in parallel for each RNA sample to exclude false-positive qPCR amplification caused by contaminating plasmid DNA. Specifically, an aliquot of the same RNA sample was processed under identical conditions but without the addition of reverse transcriptase, and the resulting no-RT product was subjected to qPCR using the same *TRAF6* primer pair. Reverse transcription of miRNA and mRNA was performed using the Mir-X miRNA First-Strand Synthesis Kit (Takara, Japan) and HiScript III RT SuperMix for qPCR (+gDNA wiper) (Vazyme Biotech, Nanjing, China), respectively. All reaction systems were prepared on ice per the manufacturer’s instructions. Forward and reverse primers for gga-miR-7b were designed using miRNA Design V1.01 software (Vazyme Biotech, Nanjing, China). qPCR was performed to detect miRNA and mRNA expression levels using the miRNA Universal SYBR qPCR Master Mix and ChamQ SYBR qPCR Master Mix (Low ROX Premixed) fluorescent quantitative kit (both from Vazyme Biotech, Nanjing, China). *U6* and *β-actin* served as the reference gene for miRNA and mRNA, respectively. Primers for mRNA were designed with Primer Premier software (v.5.00) and synthesized by Shanghai Sangon Biotech Co., Ltd. (Shanghai, China). All primer sequences are shown in Table S3.

### 2.9. Enzyme-Linked Immunosorbent Assay

The samples of blood and cell supernatant were collected and centrifuged at 1000× *g* for 20 min at 4 °C using a high-speed centrifuge. The upper-layer plasma and supernatant were collected for ELISA detection, while the lower-layer precipitates were discarded. The levels of chicken inflammatory cytokines (including IL-6, IL-8, IL-1β, and TNF-α) were detected using chicken-specific commercial ELISA kits (R&D Systems, Minneapolis, MN, USA) according to the manufacturer’s instructions. The assay range and analytical sensitivity were 20–640 pg/mL and <1.0 pg/mL for IL-1β, 1–32 pg/mL and <0.1 pg/mL for IL-6, 3.75–120 pg/mL and <0.1 pg/mL for IL-8, and 2.5–80 pg/mL and <0.1 pg/mL for TNF-α. The manufacturers indicated that these kits were developed for chicken cytokines, and no significant cross-reactivity or interference was reported.

### 2.10. Cell Apoptosis Assay

The Annexin V-FITC Cell Apoptosis Detection Kit (Beyotime, Shanghai, China) was employed to detect the apoptosis level of chicken DF-1 cells in this experiment. Chicken DF-1 cells in good growth status were evenly seeded in 6-well plates. When the cell reached 70% confluence, the cells were transfected for 24 h, followed by an additional 6 h stimulation with *E. tenella* sporozoites. The cell samples were collected and stained with Annexin V-FITC and PI staining buffer, and then the apoptosis levels were detected using a flow cytometer. The results obtained from the flow cytometer were visualized and analyzed using CytExpert software (v.2.3) (Beckman Coulter, Brea, CA, USA).

### 2.11. Western Blot Assay

Total protein was extracted from DF-1 cells using RIPA lysis buffer (200 μL/per well; containing 1% protease and phosphatase inhibitor) (Beyotime, Shanghai, China), lysed on ice for 30 min. Protein concentrations were quantified using a BCA Protein Quantification Kit (Vazyme, Nanjing, China) according to the manufacturer’s instructions, and samples were then normalized to identical concentrations using RIPA lysis buffer. Protein supernatants were mixed with 4 × SDS-PAGE loading buffer (Solarbio, Beijing, China) and denatured by heating. Equal volumes of protein samples and marker (11-245KD) (Solarbio, Beijing, China) were loaded into each well of Precast Gels Bis-Tris 4–20% (WSHT, Shanghai, China) for electrophoresis. Separated protein samples were transferred to a polyvinylidene fluoride (PVDF) membrane (Beyotime, Shanghai, China). After blocking with 5% bovine serum albumin (BSA; Solarbio, Beijing, China) at room temperature for 1 h, then the PVDF membranes were incubated in primary antibodies 4 °C for 10–12 h (Table S5). After incubation, the membrane was washed 3–4 times (room temperature) with 1 × TBST. HRP-conjugated Goat anti-Rabbit IgG (Cwbio, Taizhou, China) was used as the secondary antibody to incubate for 1 h at room temperature. An appropriate amount of ECL solution (Abbkine, Wuhan, China) was uniformly dropped onto the detection bands of the PVDF membrane for visualization and then performed using an Chemiluminescence Imaging System (Tanon 5200, Shanghai, China) for image acquisition and the results were analyzed using ImageJ software (v.1.53).

### 2.12. MiRNA Prediction and Dual-Luciferase Reporter Assay

The prediction of potential regulatory miRNAs of the *TRAF6* gene were performed using miRDB and miRanda. Subsequently, possible binding sites between gga-miR-7b and the *TRAF6* 3′ untranslated region (3′UTR) were predicted using RNAhybrid. The original 3’UTR sequence of *TRAF6* was used as the wild-type (WT) insert, while mutant (MUT) inserts were generated by mutating the predicted binding sites following the base conversion principle (Table S4). All plasmids were synthesized and verified by Sanger sequencing by Genecreate (Wuhan, China). Chicken DF-1 cells were uniformly seeded into a 24-well plate until reaching 70% confluence. Then, co-transfection of miRNA (gga-miR-7b mimic or mimic-NC) and plasmids (pmirGLO-TRAF6-WT or MUT for 24 h) occurred, and DF-1 cells were processed with a dual-luciferase reporter gene assay kit (Beyotime, Shanghai, China). The fluorescence of firefly and Renilla luciferase were detected by Multimode Plate Reader (PerkinElmer, Germany).

### 2.13. Bioinformatics Analysis of TRAF6

The physicochemical properties, hydrophilicity/hydrophobicity, transmembrane domains, and signal peptides of chicken TRAF6 proteins were predicted and analyzed using online programs including ProtParam [28], ProtScale [29], TMHMM 2.0 [30], and SignalP 4.1 [31], respectively. In addition, the phosphorylation sites, signaling pathways, and secondary and tertiary structures of chicken TRAF6 proteins were predicted using NetPhos 3.1 [32], KEGG, GOR4, and SWISS-MODEL [33] online programs, respectively. Detailed information of the relevant online programs is shown in Table S6. The amino acid sequences of the *TRAF6* genes in 24 different species of birds, mammals, and fish were retrieved from the National Center for Biotechnology Information database. Multiple sequence alignment and phylogenetic tree construction of the TRAF6 proteins from different species were performed using ESPript 3.0 and MEGA 11 [34] software, respectively.

### 2.14. Statistical Analysis

The quantitative results were processed and analyzed using the 2-ΔΔCT method [35]. All experimental data were organized using Excel 2019 software. Comparisons of differences between the two groups were performed using the independent samples *t*-test in SPSS 25.0 statistical software (SPSS Inc., Chicago, IL, USA). One-way ANOVA analysis compared the multiple groups combined with Duncan’s multiple range test. The data are presented as mean ± standard deviation, and visualization of the data was conducted using GraphPad Prism software (v.8.0.2) (San Diego, CA, USA). The correlations of the expression levels between different genes were performed using Spearman’s correlation coefficient in Origin 2024 (v.10.1) analysis software. The results were presented as the correlation coefficient r and the corresponding *p*-value. A value of 0 < *r* ≤ 1 indicates a positive correlation, while −1 ≤ *r* < 0 indicates a negative correlation. * *p* < 0.05 was considered statistically significant.

## 3. Results

### 3.1. Bioinformatic Characterization of TRAF6 in Chickens

To elucidate the structural basis of chicken TRAF6 in signaling regulation, we performed comprehensive bioinformatic analyses of its sequence and molecular features. The complete CDS region of the chicken *TRAF6* spans 1638 bp, encoding a total of 545 amino acids (Figure S2A). Hydrophilicity/hydrophobicity analysis revealed a grand average of hydropathicity (GRAVY) of −0.496. Hydropathicity reflects the overall hydrophobic or hydrophilic character of a protein based on its amino acid composition. A negative GRAVY value indicates that TRAF6 is hydrophilic protein, which supports its solubility in the cytoplasmic environment (Figure S2B). Consistently, transmembrane domain (Figure S2D) and signal peptide (Figure S2C) analyses demonstrated that TRAF6 lacks both transmembrane structure and signal peptides, defining it as a cytoplasmic protein—an essential localization for its role in intracellular signaling cascades. Post-translational modifications prediction identified 38 potential phosphorylation sites (20 serine, 14 threonine, and 4 tyrosine sites; Figure S2E). Notably, TRAF6 was predicted to be a well-characterized key regulator in the NF-κB signaling pathway (Figure S2F) using KEGG databases. The NF-κB signaling pathway serves as a core regulatory axis in the host innate immune response. The secondary structure prediction showed that the TRAF6 protein includes alpha helix (27.16%), extended strand (18.9%), and random coil (53.94%) (Figure S2G), and the tertiary structure aligned with these secondary structural features (Figure S2H).

The phylogenetic tree revealed the genetic relationships among TRAF6 amino acid sequences across 24 species, encompassing birds, mammals, and fish. The results showed that the TRAF6 of all species was clustered into two large branches. Specifically, the chicken TRAF6 sequence exhibited relatively close genetic distances with those of other avian species in the evolutionary tree, while fish formed an independent cluster (Figure S2I). Multiple sequence alignment of TRAF6 (Figure S3) further revealed avian high sequence conservation, implying conserved biological functions across avian species.

### 3.2. Pathological and Molecular Responses of Chicken Cecal Tissues After E. tenella Infection

**Figure 2:**
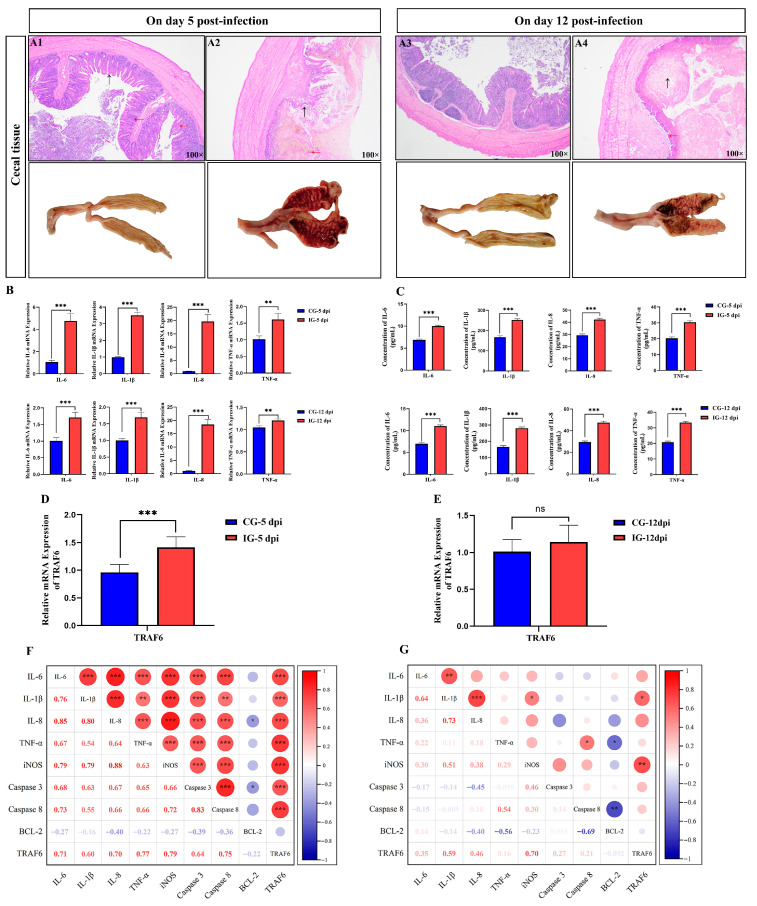
Detection of pathological and molecular responses of chicken cecal tissues to *E. tenella* infection. (**A1**–**A4**) Histopathological and histological analysis of chicken cecal tissues in control and infected group at 5 (A1 and A2) and 12 (A3 and A4) days post-infection (dpi). (**B**,**C**) The mRNA and protein concentration levels of inflammatory cytokines IL-6, IL-1β, IL-8, and TNF-α in chicken cecal tissues and serum were determined by RT-qPCR and ELISA at 5 and 12 dpi (*n* = 15). ELISA data are shown as absolute cytokine concentrations (pg/mL). The assay ranges and sensitivities of the kits are provided in the Methods section. (**D**,**E**) The relative mRNA expression levels of *TRAF6* gene in chicken cecal tissues were detected by RT-qPCR at 5 and 12 dpi (*n* = 15). (**F**,**G**) Correlation analysis between *TRAF6* and immune-related genes in chicken cecal tissues at days 5 (**F**) and 12 (**G**) after *E. tenella* infection (*n* = 15). Red indicates positive correlations while blue indicates negative. The larger the circle, the stronger the correlation was. CG-5 dpi, control group at 5 dpi; IG-5 dpi, infected group at 5 dpi; CG-12 dpi, control group at 12 dpi; IG-12 dpi, infected group at 12 dpi. ns *p* > 0.05, * *p* < 0.05, ** *p* < 0.01, *** *p* < 0.001.

To characterize host responses to *E. tenella* infection, we analyzed cecal histopathology, inflammatory cytokine expression, *TRAF6* expression pattern, and their correlations at 5 and 12 dpi. The phenotypic traits of cecal tissue showed that control cecal tissues exhibited clear and intact mucosal architectures, with a continuous and tightly arranged mucosal epithelium (Figure 2(A1), red arrows), and an adequate number of neatly aligned crypts (Figure 2(A1), black arrows). Infected ceca showed severe hemorrhage, mucosal swelling, epithelial necrosis/shedding, inflammatory cell infiltration (Figure 2(A2), red arrows), and visible oocysts at 5 dpi (Figure 2(A2), black arrows). By 12 dpi, infected ceca still displayed thickened walls, epithelial degeneration and shedding (Figure 2(A4), black arrows). At the boundary between necrotic lesions and relatively normal tissue, a large number of inflammatory cells diffusely infiltrated (Figure 2(A4), red arrows), though hemorrhagic spots were reduced, confirming persistent cecal damage across infection stages.

Additionally, relative mRNA levels of *IL-6*, *IL-1β*, *IL-8*, and *TNF-α* in infected ceca were significantly elevated (*p* < 0.05; Figure 2B or Table S7), and this upregulation persisted on 12 dpi (*p* < 0.05; Figure 2B or Table S7). The serum protein concentrations of these cytokines were also increased in infected chickens at 5 and 12 dpi (*p* < 0.05; Figure 2C), indicating sustained systemic inflammation. *TRAF6* mRNA was significantly upregulated in infected ceca on 5 dpi (*p* < 0.05; Figure 2D) but showed no significant difference between groups on 12 dpi (*p* > 0.05; Figure 2E), demonstrating *TRAF6* as an early-responsive gene to *E. tenella* infection. To further confirm *TRAF6* as a key regulatory molecule during *E. tenella* infection, we analyzed the correlation between *TRAF6* expression and relevant factors in cecal tissues. As shown in Figure 2F,G, at 5 dpi, *TRAF6* expression correlated strongly positively with pro-inflammatory cytokines (*IL-6*, *IL-1β*, *IL-8*, *TNF-α*, *iNOS*; *p* < 0.05) and apoptotic markers (*Caspase 3*, *Caspase 8*; *p* < 0.05), with no association with *BCL-2*. By 12 dpi, inflammatory cytokines remained correlated with each other but showed no significant association with *TRAF6* (*p* > 0.05). These results suggested that TRAF6 may play a regulatory role in the inflammation response to coccidial infection.

### 3.3. The Expression Pattern of TRAF6 in E. tenella Sporozoite-Infected DF-1 Cells

**Figure 3:**
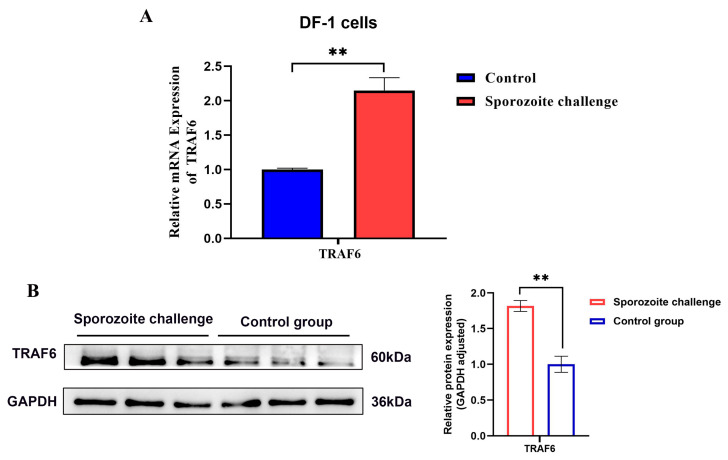
Expression of *TRAF6* in *E. tenella* sporozoite-infected DF-1 cells for 6 h. (**A**) mRNA expression (RT-qPCR); (**B**) protein expression (Western blot) (*n* = 3). ** *p* < 0.01. The original western blot images can be found in the Supplementary Material.

To verify this, we further analyzed the expression kinetics of *TRAF6* in vitro infection. RT-qPCR results confirmed that *TRAF6* mRNA level was significantly upregulated in *E. tenella* sporozoite-invaded DF-1 cells (*p* < 0.01; Figure 3A). Consistent with the transcriptional level, Western blot analysis confirmed that TRAF6 protein level accumulated dramatically during sporozoite-infected DF-1 cells (*p* < 0.01; Figure 3B). These results also indicated that TRAF6 may be involved in the host cell response mediated by coccidian infections.

### 3.4. The Construction of TRAF6 Overexpression and Knockdown Vectors and Validation of Transfection Efficiency in Chicken DF-1 Cells

To evaluate the possible effect of *TRAF6* during coccidia infection, gain-of-function and loss-of-function experiments were conducted. The full-length CDS region of *TRAF6* (1638 bp) was cloned between the *BamHI* and *EcoRI* restriction sites of the pcDNA3.1 empty vector to construct the recombinant overexpression plasmid pcDNA3.1-TRAF6 (Figure S4A). In addition, to verify transfection delivery in DF-1 cells, pcDNA3.1-GFP plasmid and a FAM- labeled siRNA oligonucleotide were separately transfected, and fluorescence was observed by inverted fluorescence microscopy at 24 h post-transfection. Green fluorescence was visualized by inverted fluorescence microscopy, confirming successful transfection efficiency of the pcDNA3.1 and siRNA (Figure S4B). As shown in Figure S4C,E, we observed that transfection with pcDNA3.1-TRAF6 significantly elevated both mRNA and protein expression levels of TRAF6 in DF-1 cells (*p* < 0.05). To exclude the possibility that the elevated RT-qPCR signal resulted from residual plasmid DNA contamination, a no-RT control was included in the analysis, and no detectable amplification was observed in the absence of reverse transcription (Figure S5). Meanwhile, we also designed four small interfering RNA sequences for targeting *TRAF6*. The siRNA-517 had the best interference effect (inhibition rate: ~60%), and significantly suppressed both mRNA and protein expression levels of TRAF6 in DF-1 cells (*p* < 0.05, Figure S4D,F), so siRNA-517 was selected for subsequent functional analyses.

### 3.5. TRAF6 Promotes Inflammation and Apoptosis in E. tenella-Infected DF-1 Cells by Inducing NF-κB Signaling Pathway Activation

**Figure 4:**
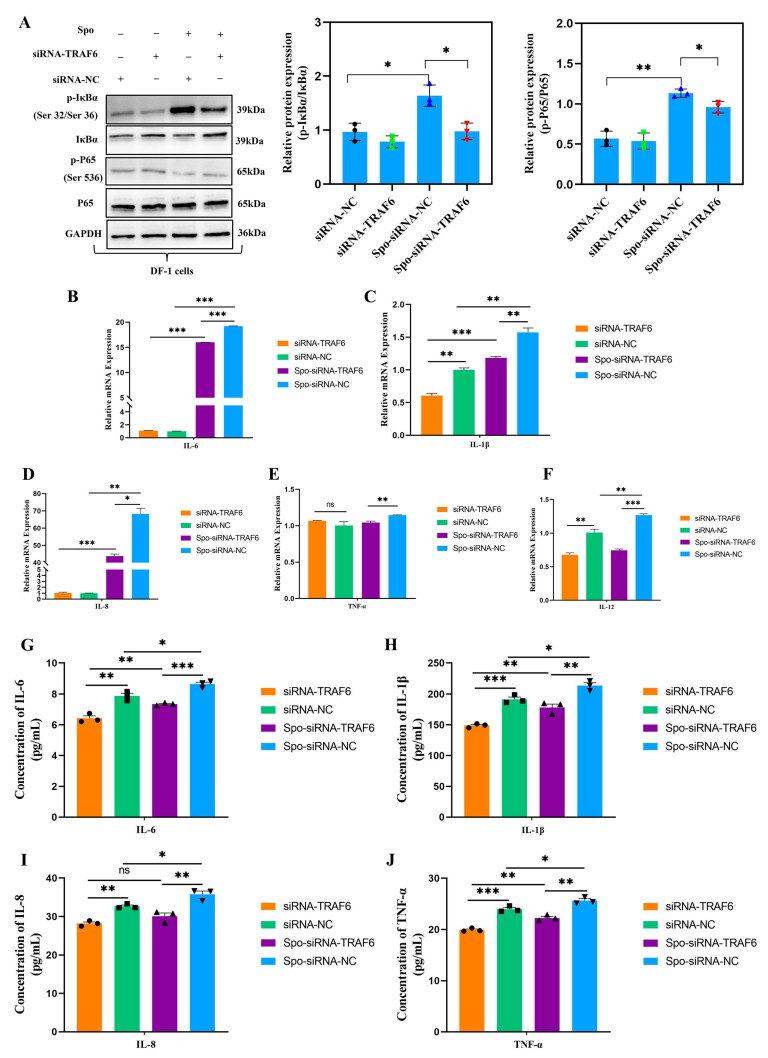
The effects of *TRAF6* knockdown on NF-κB signaling pathway, and production of inflammation cytokines in *E. tenella* sporozoite-infected chicken DF-1 cells. DF-1 cells were transfected for 24 h and then stimulated with *E. tenella* sporozoites for an additional 6 h. (**A**) Western blot analysis of changes in abundance and phosphorylation levels of key NF-κB signaling proteins following *TRAF6* knockdown in DF-1 cells (*n* = 3). (**B**–**F**) RT-qPCR was conducted to detect the mRNA expression levels of inflammatory cytokines *IL-6*, *IL-1β*, *IL-8*, *TNF-α*, and *IL-12* in DF-1 cells transfected with *TRAF6* siRNA (*n* = 3). *β-actin* was used as the reference gene. (**G**–**J**) The protein secretion levels of inflammatory cytokines in supernatants of DF-1 cells were measured by ELISA (*n* = 3). ELISA data are shown as absolute cytokine concentrations (pg/mL). The assay ranges and sensitivities of the kits are provided in the Methods section. ns *p* > 0.05, * *p* < 0.05, ** *p* < 0.01, *** *p* < 0.001. The original western blot images can be found in the Supplementary Material.

**Figure 5:**
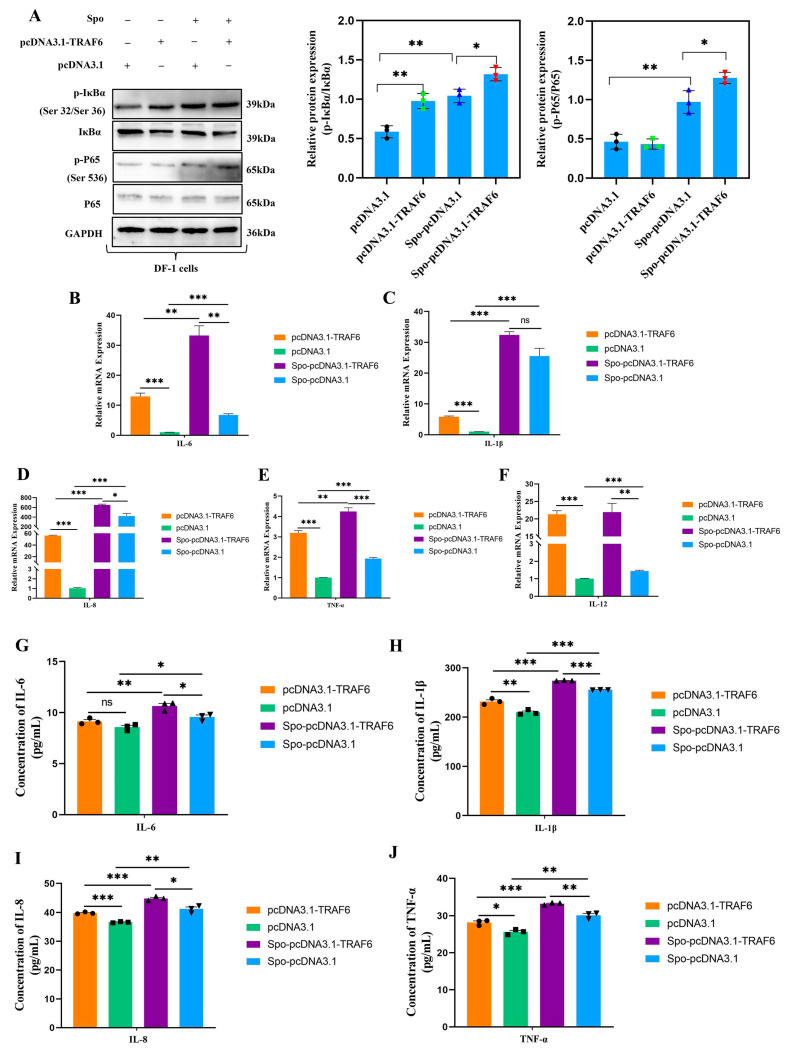
The effects of *TRAF6* overexpression on NF-κB signaling pathway, and the production of inflammation cytokines in *E. tenella* sporozoite-infected chicken DF-1 cells. DF-1 cells were transfected for 24 h and then stimulated with *E. tenella* sporozoites for an additional 6 h. (**A**) Western blot analysis of changes in abundance and phosphorylation levels of key NF-κB signaling proteins following *TRAF6* overexpression in DF-1 cells (*n* = 3). (**B**–**F**) The mRNA expression levels of inflammatory cytokines *IL-6*, *IL-1β*, *IL-8*, *TNF-α*, and *IL-12* in DF-1 cells transfected with pcDNA3.1-TRAF6 were analyzed by RT-qPCR (*n* = 3). *β-actin* was used as the reference gene. (**G**–**J**) The protein secretion levels of inflammatory cytokines in supernatants of DF-1 cells were measured by ELISA (*n* = 3). ELISA data are shown as absolute cytokine concentrations (pg/mL). The assay ranges and sensitivities of the kits are provided in the Methods section. ns *p* > 0.05, * *p* < 0.05, ** *p* < 0.01, *** *p* < 0.001. The original western blot images can be found in the Supplementary Material.

Based on the above findings, we focused on *TRAF6* in the present study and explored its functions through gain-of-function and loss-of-function experiments in the *E. tenella* infection process. Our results demonstrated that compared with the siRNA-NC, *TRAF6* knockdown (using specific siRNA) moderately reduced *E. tenella*-induced NF-κB signaling activation, as indicated by decreased phosphorylation protein levels of IκBα and P65 (*p* < 0.05; Figure 4A). RT-qPCR and ELISA analyses showed that *TRAF6* knockdown moderately reduced the mRNA expression and protein secretion of pro-inflammatory cytokines (IL-6, IL-8, IL-1β, TNF-α, and IL-12) in infected cells (*p* < 0.05; Figure 4B–J). To further clarify the function of *TRAF6* during *E. tenella* infection, *TRAF6* plasmid was used to enhance its expression. The results demonstrated that *TRAF6* overexpression enhanced NF-κB activation in *E. tenella*-infected DF-1 cells, as evidenced by increased p-IκBα and p-p65 protein levels (*p* < 0.05; Figure 5A). This was accompanied by the upregulation of pro-inflammatory cytokine (IL-6, IL-8, IL-1β, TNF-α, and IL-12) mRNA expression and protein secretion (*p* < 0.05; Figure 5B–J).

**Figure 6:**
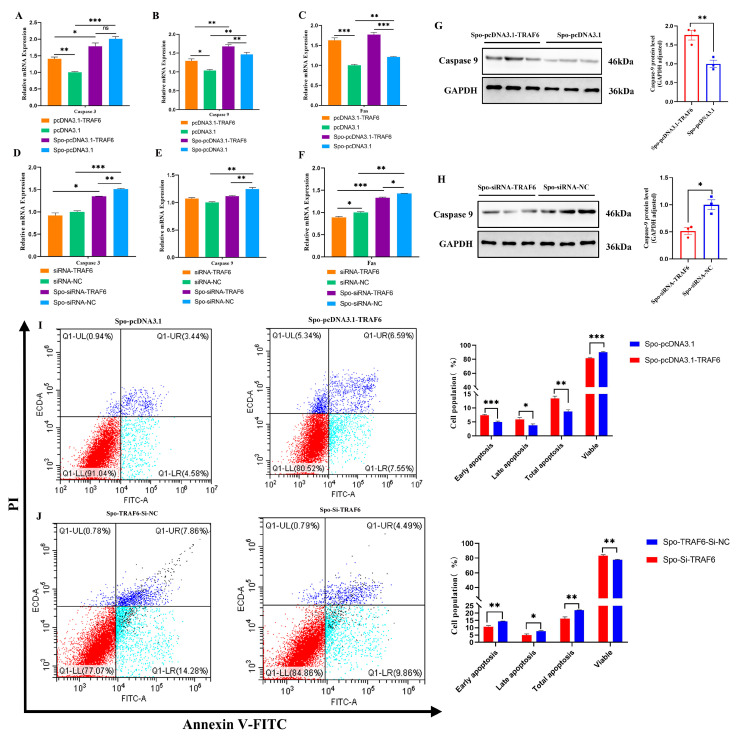
Effect of *TRAF6* on apoptosis in sporozoite-infected chicken DF-1 cells. DF-1 cells were transfected for 24 h and then stimulated with *E. tenella* sporozoites for an additional 6 h. (**A**–**C**) The mRNA expression levels of *Caspase 3* (**A**), *Caspase 9* (**B**), and *Fas* (**C**) in DF-1 cells overexpressed *TRAF6* (*n* = 3). (**D**–**F**) The mRNA expression levels of *Caspase 3* (**D**), *Caspase 9* (**E**), and *Fas* (**F**) in DF-1 cells interfered *TRAF6* (*n* = 3). *β-actin* was used as the reference gene. (**G**,**H**) The protein level of Caspase-9 in sporozoite-infected DF-1 cells transfected with *TRAF6* overexpression or interference (*n* = 3). (**I**,**J**) Cell apoptosis rates of sporozoite-infected DF-1 cells after transfection with *TRAF6* overexpression or interference (*n* = 3). ns *p* > 0.05, * *p* < 0.05, ** *p* < 0.01, *** *p* < 0.001.The original western blot images can be found in the Supplementary Material.

In addition to its effects on inflammatory responses, *TRAF6* also modulated apoptosis in *E. tenella*-infected DF-1 cells. *TRAF6* knockdown downregulated the mRNA levels of *Caspase 3*, *Caspase 9*, and *Fas* (*p* < 0.05; Figure 6D–F), and reduced Caspase 9 protein abundance in *E. tenella*-infected DF-1 cells (*p* < 0.05; Figure 6H). Consistently, flow cytometry confirmed that *TRAF6* knockdown effectively inhibited *E. tenella*-induced DF-1 cells apoptosis (*p* < 0.05; Figure 6J). In contrast, *TRAF6* overexpression upregulated the mRNA levels of *Caspase 3*, *Caspase 9*, and *Fas*, (*p* < 0.05; Figure 6A–C) and increased Caspase 9 protein abundance (*p* < 0.05; Figure 6G), with flow cytometry confirming a marked increase in the apoptotic rate of *E. tenella*-infected DF-1 cells (*p* < 0.05; Figure 6I). Collectively, these results demonstrate that *E. tenella* infection-induced upregulation of *TRAF6* releases the “brake” on the NF-κB signaling pathway, leading to its excessive activation and the subsequent promotion of inflammatory responses and apoptotic damage in host cells.

### 3.6. TRAF6 Is Targeted and Regulated by gga-miR-7b

**Figure 7:**
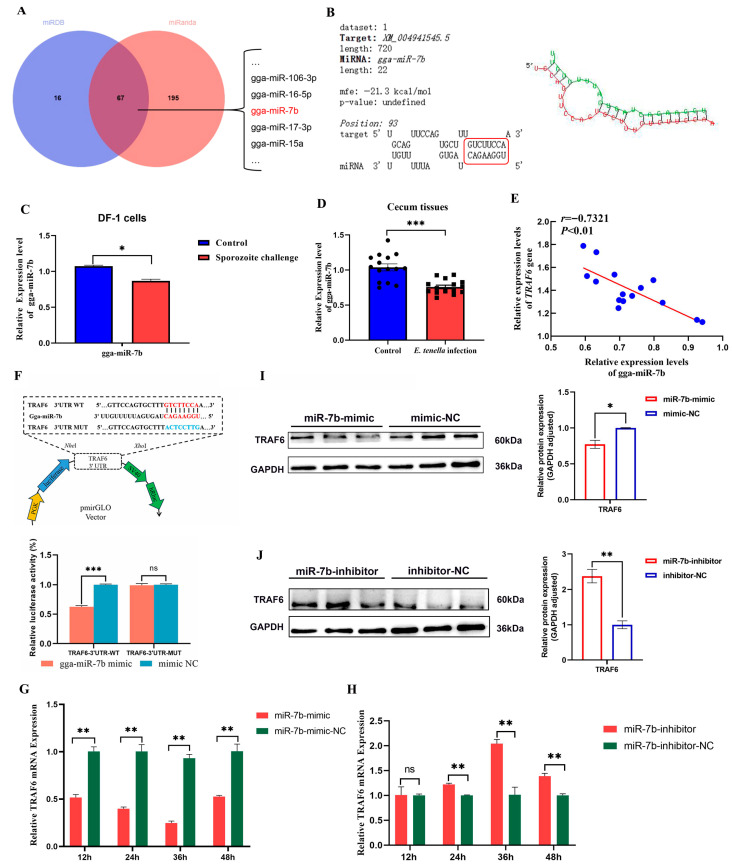
*TRAF6* is a gga-miR-7b target gene. (**A**) miRDB and miRanda databases were used to predict potential regulatory miRNAs of the *TRAF6* gene. (**B**) Predicted results of potential targeted binding sites between gga-miR-7b and the *TRAF6* gene via RNAhybrid (the sequence information is shown in the red box). (**C**,**D**) Expression levels of gga-miR-7b in chicken cecal tissues (*n* = 15) and DF-1 cells (*n* = 3) after *E. tenella* infection. (**E**) The correlation coefficient (r) between *TRAF6* mRNA and gga-miR-7b expression in *E. tenella*-infected chicken cecal tissues was verified by Spearman’s rank correlation analysis (*n* = 15). (**F**) Results of the dual-luciferase reporter assay for gga-miR-7b and its target gene *TRAF6* in DF-1 cells at 24 h post-transfection (*n* = 6). (**G**,**H**) Effect of gga-miR-7b mimic (**G**) and inhibitor (**H**) on *TRAF6* mRNA expression levels in chicken DF-1 cells at 12, 24, 36, and 48 h post-transfection (*n* = 3). (**I**,**J**) Effects of gga-miR-7b mimic (**I**) and inhibitor (**J**) on TRAF6 protein expression levels in chicken DF-1 cells at 24 h post-transfection (*n* = 3). ns *p* > 0.05, * *p* < 0.05, ** *p* < 0.01, *** *p* < 0.001.The original western blot images can be found in the Supplementary Material.

To explore the potential regulatory molecules of chicken *TRAF6* during *E. tenella* infection, we jointly screened 67 key miRNAs using two prediction databases, miRDB and miRanda (Figure 7A). Notably, among these miRNAs, gga-miR-7b has been predicted in our previous study to be significantly differentially expressed during coccidial infection [36]. Furthermore, we identified a binding site between the seed sequence of gga-miR-7b and the 3’UTR region of *TRAF6*, with a free energy of −21.3 kcal/mol, indicating a stable targeted binding interaction between the two (Figure 7B). RT-qPCR analysis showed that the expression level of gga-miR-7b was significantly downregulated in both *E. tenella*-infected cecal tissues and sporozoite-infected DF-1 cells (*p* < 0.05, Figure 7C,D), and showed a significant negative correlation with the expression of *TRAF6* (*p* < 0.05, Figure 7E). Subsequently, we constructed WT and MUT recombinant vectors of the *TRAF6* 3′UTR and performed a dual-luciferase reporter assay (Figure S6). The relative luciferase activity in the gga-miR-7b mimic + TRAF6-3’UTR-WT group was significantly lower than that in the mimic NC + TRAF6-3’UTR-WT group (*p* < 0.05, Figure 7F). In contrast, no significant difference in relative luciferase activity was observed between the gga-miR-7b mimic and mimic NC in the TRAF6-3’UTR-MUT group (*p* > 0.05, Figure 7F). These results demonstrated that the *TRAF6* 3′UTR region can be targeted by gga-miR-7b, with “GTCTTCCA” being the binding site between them. We further investigated how gga-miR-7b regulates the expression levels of the TRAF6 using RT-qPCR and Western blot. The results showed that the overexpression of gga-miR-7b significantly inhibited the mRNA and protein expression level of TRAF6 in DF-1 cells (*p* < 0.05, Figure 7G,I). In contrast, for gga-miR-7b inhibition, the expression level of TRAF6 in cells was significantly higher than that of the inhibitor-NC group (*p* < 0.05, Figure 7H,J).

### 3.7. Upregulation of TRAF6 Rescues the Effect of gga-miR-7b on the Inflammatory Response in Sporozoites-Induced DF-1 Cells by Activating NF-κB Signaling Pathway

**Figure 8:**
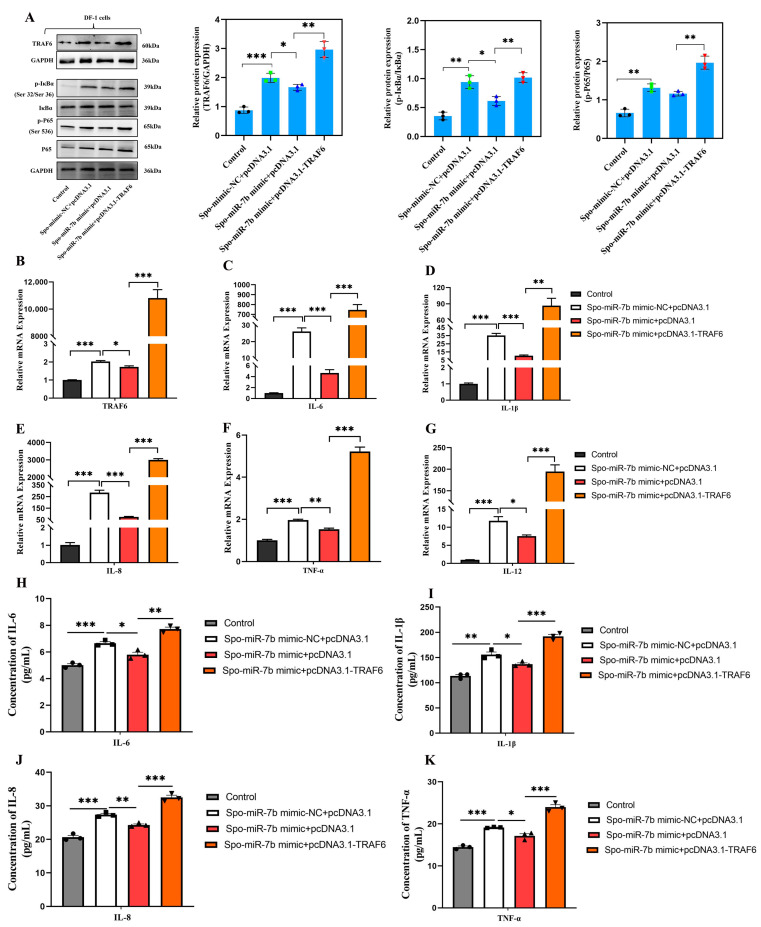
Upregulation of *TRAF6* rescues the effect of gga-miR-7b on the inflammatory response in sporozoites-induced DF-1 cells by activating NF-κB signaling pathway. (**A**) Changes in NF-κB signaling proteins’ abundance and TRAF6 protein expression after co-transfection with gga-miR-7b mimic and pcDNA3.1-TRAF6 (*n* = 3). (**B**–**G**) RT-qPCR was performed to detect mRNA expression levels of *TRAF6*, *IL-6*, *IL-1β*, *IL-8*, *TNF-α*, and *IL-12* in chicken DF-1 cells co-transfected with gga-miR-7b and *TRAF6* overexpression vectors. *β-actin* was used as internal reference gene (*n* = 3). (**H**–**K**) ELISA was conducted to determine protein secretion levels of inflammatory cytokines in the supernatant of chicken DF-1 cells (*n* = 3). ELISA data are shown as absolute cytokine concentrations (pg/mL). Assay ranges and sensitivities of kits are provided in Methods section. * *p* < 0.05, ** *p* < 0.01, *** *p* < 0.001.The original western blot images can be found in the Supplementary Material.

Subsequently, rescue experiments (co-transfection strategy) were performed to directly confirm that gga-miR-7b can target *TRAF6* to regulate the inflammatory response induced by *E. tenella* infection through the NF-κB signaling pathway. As expected, *E. tenella* infection significantly activated NF-κB signaling, as indicated by the elevated phosphorylation of IκBα and p65 (Figure 8A), and concomitantly increased inflammatory cytokine production compared with the uninfected group (Figure 8C–K). Meanwhile, gga-miR-7b mimic transfection markedly suppressed *E. tenella*-induced NF-κB activation and inflammatory cytokine release, demonstrating a clear anti-inflammatory effect (Figure 8A,C–K). Notably, this inhibitory effect was significantly reversed by co-transfection with the TRAF6 overexpression plasmid. *TRAF6* overexpression restored TRAF6 protein abundance, along with the phosphorylation levels of IκBα and p65 and inflammatory cytokine production that had been suppressed by gga-miR-7b mimic, thereby rescuing the inflammatory phenotype induced by *E. tenella* infection (Figure 8A–K). Collectively, these findings provide further evidence that gga-miR-7b attenuates *E. tenella*-triggered inflammation, at least in part, through targeting TRAF6 and inhibiting NF-κB signaling.

## 4. Discussion

In chickens, coccidial infection can severely induce host inflammatory responses, which may cause intestinal mucosal inflammatory damage, intestinal wall swelling, and nutritional metabolism disorders, resulting in substantial annual losses to the global poultry industry. Therefore, the prevention and control of coccidiosis have become particularly important. Relevant studies have demonstrated that TRAF6, as an adaptor protein, is widely involved in the transduction of multiple inflammatory signaling pathways and plays a crucial role in various inflammatory diseases, such as pulmonary inflammation [37], inflammatory bowel disease [38], and hepatic inflammation [39], thereby showing potential as a therapeutic target for inflammatory diseases. However, whether it can serve as an effective marker for the future diagnosis, prevention, and control of coccidiosis remains to be explored.

The infection process of coccidia essentially corresponds to a complete life cycle of the parasite [40,41]. Following coccidial infection, oocysts begin to shed on the day 5 PI and reach a shedding peak between days 7–9 PI, after which they gradually shed until complete elimination [42]. Therefore, we respectively investigated the potential relationship between the *TRAF6* and the inflammatory and apoptosis factors in chicken cecal tissues at days 5 and 12 PI, the early and late periods of *E. tenella* infection. In our research, the expression levels of *TRAF6* in chicken cecal tissues were detected to be extremely significantly upregulated at day 5 PI, and showed significant positive correlations with *IL-6*, *IL-1β*, *IL-8*, *TNF-α*, *iNOS*, *Caspase 3* and *Caspase 8*, whereas no significant correlations were observed at day 12 PI.

Initially, members of the TRAF family were identified as signaling adaptor proteins that directly bind to members of the TNFR superfamily [43,44]. With the advancement of research, TRAF molecules have been shown to broadly participate in the signal transduction of various adaptive immune receptors, innate immune receptors, and cytokine receptors [45,46]. Cytokine receptors that can directly or indirectly recruit TRAF proteins include those for IL-1β, IL-2, IL-6, IL-17, IL-18, IL-33, type I interferons (IFNs), and type III IFNs, among others [16,45,46]. Thus, TRAF molecules play an important role in hosts’ immune responses against pathogenic infections. Therefore, we also hypothesized that TRAF6 might play an important regulatory role in the process of *E. tenella* infection to the host.

Numerous studies have extensively explored the regulatory roles of different functional genes by constructing knockout or overexpression methods in vitro [36,47]. In this study, overexpression and knockdown vectors of the *TRAF6* were synthesized in vitro. RT-qPCR and Western blot assays demonstrated that these constructs could significantly promote or inhibit the expression level of TRAF6 in chicken DF-1 cells, laying a foundation for subsequent investigations. Among members of the TRAF family, TRAF6 has been extensively studied in inflammatory responses and is now known to play crucial roles in activating key signaling pathways such as the NF-κB, MAPK (JNK1/2, ERK1/2, and p38), and IRF (IRF3, IRF4, IRF5, and IRF7) pathways [48,49,50]. Additionally, studies have also demonstrated that TRAF6 is critically involved in various inflammatory diseases in humans or mice, including pulmonary inflammation [48], cardiovascular inflammation [51], inflammatory bowel disease [17], hepatic inflammation [52], pancreatitis [53], and diabetes. These studies indicated that TRAF6 is a key regulator of immune homeostasis and has potential as a therapeutic target for inflammatory diseases. However, research on the function of TRAF6 in poultry diseases and inflammatory responses remains limited. Recent studies have focused on the identification and expression profiling of TRAF6 during viral diseases and pathogenic microbial infections. For example, Li et al. [54] reported that the mRNA levels of *TRAF6*, *TLR7*, and *IKKβ* in chicken kidney tissues were significantly upregulated after infection with infectious bronchitis virus (IBV). Similarly, Xu et al. [55] found that *Reuterin*, a cellular metabolite, can exert anti-inflammatory effects on LPS-stimulated chicken macrophages by downregulating the expression of TLR4/MyD88/TRAF6. Our results demonstrate that *E. tenella* infection-induced upregulation of *TRAF6* releases the “brake” on the NF-κB signaling pathway, leading to its excessive activation and the subsequent promotion of inflammatory responses in host cells. It is important to note that TRAF6 signaling may represent a double-edged mechanism during *E. tenella* infection. Although suppression of TRAF6 may alleviate excessive inflammation and apoptosis, it could also weaken host antiparasitic defense and thereby affect parasite burden, clearance, and transmission. Therefore, the in vivo consequences of targeting TRAF6 require further investigation, particularly with respect to the balance between reducing immunopathology and maintaining effective parasite control.

The precise regulation of apoptosis serves as a crucial immune defense mechanism employed by the host organism [56]. The invasion and developmental process of pathogens into the host is accompanied by host cell apoptosis [57]. In addition to the inflammatory phenotype, our flow-cytometry data further demonstrated that *TRAF6* modulates the apoptotic response of *E. tenella*-infected DF-1 cells. *TRAF6* overexpression increased the proportion of apoptotic cells, whereas *TRAF6* knockdown produced the opposite effect, indicating that TRAF6 contributes not only to inflammatory activation but also to infection-associated cell apoptotic injury. Notably, these phenotypic changes were consistent with the expression patterns of apoptosis-related molecules. Caspase 9, a key initiator of the intrinsic apoptotic pathway, and Fas, a representative death-receptor signal of the extrinsic pathway, were both upregulated by TRAF6 overexpression and downregulated by TRAF6 knockdown, while Caspase 3, the major executioner caspase, showed the same trend. These findings suggest that TRAF6 enhances *E. tenella*-induced apoptosis in DF-1 cells. Accordingly, in this study, we revealed a novel function for *TRAF6*, namely, the regulation of the inflammatory and apoptosis responses in the chicken during coccidial infection.

miRNAs are a class of small, endogenous, and highly conserved single-stranded non-coding RNA molecules, with a length of only 18–25 nucleotides [58]. Studies have shown that up to 60% of protein-coding genes may be regulated by miRNAs to some extent [59]. MiRNAs core mechanism of action act mainly by binding to the 3′UTR region of target mRNAs, thereby inducing their degradation and inhibiting their translation [60]. Sun et al. [58]. found that gga-miR-1306-5p can target the *Tollip* gene to regulate the host’s inflammatory response against *Salmonella* infection. Another study demonstrated that *IRS2* can alleviate lipogenesis, oxidative stress, inflammation, and apoptosis in chicken hepatocytes by mediating the PI3K/Akt pathway, and this effect can be regulated by gga-miR-27b-5p [61]. Also, prediction results suggested that there is a binding site between the chicken TRAF6 and gga-miR-7b. Dual-luciferase reporter, RT-qPCR, and Western blot analyses confirmed that TRAF6 is a direct target of gga-miR-7b and is negatively regulated by gga-miR-7b. Furthermore, rescue experiments confirmed that gga-miR-7b exerts its regulatory effect on NF-κB pathway activation and inflammatory responses by directly targeting *TRAF6* during *E. tenella* infection. Although several comparisons reached statistical significance, the magnitude of change in some readouts was relatively modest. Therefore, these results should be interpreted cautiously in terms of biological significance. In the present study, we relied primarily on the overall consistency across multiple independent assays, including pathway activation, cytokine measurements, apoptosis-related markers, and rescue experiments, rather than on any single small-amplitude change, to support the involvement of the gga-miR-7b/TRAF6/NF-κB axis in the response to *E. tenella* infection. It is important to note that discrepancies between mRNA expression and protein levels are frequently observed in inflammatory studies, because they reflect different layers of regulation, including transcriptional dynamics, post-transcriptional control, translation efficiency, and protein secretion kinetics [62,63,64]. Therefore, mRNA abundance does not always directly correspond to the amount of secreted protein measured at a given time point. Such differences do not necessarily weaken the biological interpretation, especially when the overall direction of change remains comparable across independent assays.

Although cecal epithelial cells are the natural target of *E. tenella*, DF-1 cells were used here as a practical chicken-derived mechanistic model because they are well established for sporozoite–host interaction studies [27,65,66] and are more amenable to the repeated transfection and rescue experiments required in this work. By contrast, primary cecal epithelial cultures remain technically demanding and short-lived, which limits their utility for stable and reproducible gain and loss-of-function analyses [67,68]. In addition, macrophages mainly represent professional immune effector cells rather than the principal epithelial niche of *E. tenella* infection, and their use would address a somewhat different biological question. Therefore, DF-1 cells were selected in this study as a suitable in vitro system for dissecting the cell-autonomous regulatory mechanism of the gga-miR-7b/TRAF6/NF-κB axis. However, these findings should be further validated in vivo and, where feasible, in more physiologically relevant epithelial models.

## 5. Conclusions

In conclusion, our study has uncovered that TRAF6 promotes inflammation and apoptosis in *E. tenella*-infected DF-1 cells by inducing NF-κB signaling pathway activation. Further, this regulatory cascade is specifically targeted and modulated by the key miRNA (gga-miR-7b).

## Data Availability

The original contributions presented in this study are included in the article/Supplementary Material. Further inquiries can be directed to the corresponding author.

## Supplementary Materials

The following supporting information can be downloaded at: https://www.mdpi.com/article/10.3390/biom16050655/s1, Table S1: The small interference RNA (siRNAs) sequence information of *TRAF6* gene; Table S2: Sequences of gga-miR-7b mimic, inhibitor and corresponding negative controls; Table S3: PCR primer list; Table S4: Insert fragments of wild-type and mutant plasmids; Table S5: Information of antibodies; Table S6: Software of bioinformatics analysis; Table S7: The quantitative data of *IL-6*, *IL-1β*, *IL-8*, *TNF-α*, and *TRAF6* in chicken cecal tissues on 5 and 12 dpi; Supplementary Word: The information of the *TRAF6* CDS sequences; Figure S1: CCK-8 analysis was performed to evaluate the cell viability after the transfections. Figure S2: Bioinformatics characterization of chicken TRAF6; Figure S3: Multiple alignment analysis of TRAF6 amino acid sequence in different species; Figure S4: Validation of *TRAF6* overexpression and knockdown efficiency; Figure S5: No-RT control validation for *TRAF6* overexpression; Figure S6; Sanger sequencing chromatograms of wild-type and mutant *TRAF6* 3′UTR recombinant plasmids; Figures S7–S13: Original Western blot images corresponding to main text.

## Abbreviations

The following abbreviations are used in this manuscript:

*E. tenella*	*Eimeria tenella*
TRAF6	Tumor necrosis factor receptor (TNFR)-associated factor 6
Gga-miR-7b	Gallus gallus microRNA-7b
MiRNA	MicroRNA
NF-κB	Nuclear factor kappaB
H&E	Hematoxylin-eosin
DF-1 cells	Chicken embryo fibroblast cell line
ELISA	Enzyme-linked immunosorbent assay
RT-qPCR	Real-time quantitative PCR
NC	Negative control
IL	Interleukin
CDS	Coding sequence
3’UTR	3′-untranslated region
