# Supplementary material for: TRAF6, a gga-miR-7b Target, Promotes Eimeria tenella-Induced Inflammation and Apoptosis in Chickens by Activating NF-κB Pathway
Source: Biomolecules. 2026 Apr 28;16(5):655. doi: 10.3390/biom16050655 (PMC13204713; doi:10.3390/biom16050655)
Supplement: Supplementary file 1 [file biomolecules-16-00655-s001.zip › Supplementary Materials—revised.pdf]

Table S1. The small interference RNA (siRNAs) sequence information of *TRAF6* gene

Gene name	Sequence name	Primer information（5'-3'）
TRAF6	siR-TRAF6-490	GAAGAUGGAGCUGAGACAUTT
		AUGUCUCAGCUCCAUCUUCTT
	siR-TRAF6-517	GCACCUGUUGCAUUGUGAUTT
		AUCACAAUGCAACUGGUGCTT
	siR-TRAF6-745	GAUGCCUAACCAUUAUGAUTT
		AUCAUAAUGGUUAGGCAUCTT
	siR-TRAF6-868	GGUUCACAUGAGAAUGAUGTT
		CAUCAUUCUCAUGUGAACCTT
Negative NC	siRNA-FAM	UUCUCCGAACGUGUCACGUTT
		ACGUGACACGUUCGGAGAATT
	siRNA-NC	UUCUCCGAACGUGUCACGUTT
		ACGUGACACGUUCGGAGAATT

Table S2. Sequences of gga-miR-7b mimic, inhibitor and corresponding negative controls

Gene name	Sequence name	Primer information（5'-3'）
gga-miR-7b	mimic	UGGAAGACUAGUGAUUUUGUU
		AACAAAAAUCACUAGUCUUCCA
	mimic-NC	UUUGUACUACACAAAAGUACUG
		CAGUACUUUUGUGUAGUACAAA
	inhibitor	AACAAAAAUCACUAGUCUUCCA
	inhibitor-NC	CAGUACUUUUGUGUAGUACAAA

Table S3. PCR primer list

Gene name	Primer sequence（5'-3'）	Product size (bp)	Annealing (°C)
IL-6	F: AAATCCCTCCTCGCCAATCT<br>R: CCTCACGGTCTTCTCCATAAA	105	60
IL-1β	F: GGTCAACATCGCCACCTACA<br>R: CATACGAGATGGAAACCAGCAA	86	60
IL-8	F: AGTTCATCCACCCTAAATCC<br>R: CACACCTCTCTTCCATCCTT	106	60
TNF-a	F: GCCCTTCCTGTAACCAGATG<br>R: ACACGACAGCCAAGTCAACG	71	60
IL-12	F: CCTGCCTGTCTGCTAAGACC<br>R: ATCATTTGCCCATTGGAGTC	82	60
iNOS	F: CCTGGGTTTCAGAAGTGGC<br>R: CCTGGAGGTCCTGGAAGAGT	82	60
Caspase-3	F: TGGCCCTCTTGAACTGAAAG<br>R: TCCACTGTCTGCTTCAATACC	139	60
Caspase-8	F: CATCTGTGGCACCCGATTCTCTG<br>R: CTTCTGAGTTCTGGCACTGCTTCC	148	60
Caspase-9	F: GCGATTCCTTTCCAGGCTCC<br>R: CACGAGCCACTCACCTTGTC	140	60
Fas	F: TCCACCTGCTCCTCGTCATT<br>R: GTGCAGTGTGTGTGGGAACT	78	60
BCL-2	F: AAGCAAGCGTGACAAC<br>R: ATCATAGGCTGCACATAC	127	60
TRAF6	F: CCATGTGTCACGTGCCCCGTATAA<br>R: TGTCTCACCAGACGACCTTCCAATT	90	51
β-actin	F: CCTGAACCTCTCATTGCCA<br>R: GAGAAATTGTGCGTGACATCA	152	60
U6	F: GGAACGATACAGAGAAGATTAGC<br>R: TGGAACGCTTCACGAATTTGCG	/	60
gga-miR-7b	F: GCGCGTGGAAGACTAGTGATT<br>R: AGTGCAGGGTCCGAGGTATT	/	60

Table S4. Insert fragments of wild-type and mutant plasmids

Insert fragments	Sequence (5' to 3')
TRAF6-WT	GTATTAATTTCTCCTGCCCTGAAACATAAATGTTAGTGACACAT TAATTTATGTGTTTGTTCCAGTTCCCTTATTCTTTCCCCACCTTT AACTGCAGTTCCAGTGCTTTGTCTTCCAAATAAGAACAGCGTC TTCAGCCAATGGAAAAAACAGAAGTCTGGATTGGTCCCACCT CACTGT
TRAF6-MUT	GTATTAATTTCTCCTGCCCTGAAACATAAATGTTAGTGACACAT TAATTTATGTGTTTGTTTCAGTTCCCTTATTCTTTCCCCACCTTT AACTGCAGTTTCAGTGCTTTACTCCTTGAATAAGAACAGCGTT TTCAGCCAATGGAAAAAACAGAAGTCTGGATTGGTCCCACCT CACTGT

Table S5. Information of antibodies

Antibody	Company	Dilution ratio
GAPDH	HUABIO	1:10000
p-IκBα	Abmart	1:1000
IκBα	Abmart	1:1000
p-P65	Abmart	1:1000
P65	Abmart	1:1000
TRAF6	HUABIO	1:1000
Caspase-9	Abmart	1:1000
Goat Anti-Rabbit IgG, HRP Conjugated	CWBIO	1:10000

Table S6. Software of bioinformatics analysis

Online software	Website information	Function prediction
ProtParam	https://web.expasy.org/protparam/	Physicochemical properties
ProtScale	https: //web.expasy.org/protscale/	Hydrophilicity/hydrophobicity
TMHMM 2.0	https://services.healthtech.dtu.dk/service.php?TMHMM-2.0	Transmembrane domains
SignalP 4.1	https://services.healthtech.dtu.dk/services/SignalP-4.1/	Signal peptides
NetPhos-3.1	https://services.healthtech.dtu.dk/services/NetPhos-3.1/	Phosphorylation sites
KEGG	https://www.genome.jp/kegg/	Signaling pathways
GOR4	https://npsa-prabi.ibcp.fr/cgi-bin/npsa_automat.pl?page=npsa_gor4.html	Secondary structures
SWISS-MODEL	https://swissmodel.expasy.org/	Tertiary structures

Supplementary Word: The information of the TRAF6 CDS sequences

TRAF6 name: TNF receptor associated factor 6

Gene ID: 423163

Species: *Gallus gallus* (chicken)

Definition predicted: Gallus gallus TNF receptor associated factor 6 (TRAF6), transcript variant X1, mRNA.

NCBI Reference Sequence: XM_004941548.5

Information of CDS sequence: 1638bp

ATGAGCTTGCTACACAGTGATAGCAGCTGTGGAGGCCGAGACTTGGATAGTGGCT

GCTGCACAGCCATGGCCAGTGCCTGCAGCGGGGGAGCAAAAGAAGACAGTGTGA

GTGTCGGCAGTGGGACTGGCAACCCGCCCAGCTCCTTCACAGAGGAGACGCAGG

GATACGATGTGGAGTTCGATCCGCCCTTGGAAAGTAAATACGAGTGCCCGATCTGT

TTGATGGCCCTTCGGGAAGCAGTGCAGACGCCATGCGGGCACCGTTTCTGCAAAG

GCTGCATTGTCAAATCAATAAGAGATGCAGGTCACAAATGTCCAGTAGACAACGA

AATTCTACTTGAAAATCAACTTTTTCCAGACAACTTCGCTAAACGGGAAATCCTTT

CATTAACTGTTAAGTGTCCCAACAAAGGCTGCTGTTTGAAGATGGAGCTGAGACAT

TTAGAGGAGCACCAGTTGCATTGTGATTTCACCACTGTGGAATGCCCACAGTGCCA

AGGAGCCTTTCAGAAGAACCACCTCAAAGAGCACATGACACAGGAGTGTCCAAG

GCGTCAAGTCTGCTGTCCAAACTGTGCCACATCCATGGCTTATGAAGATAAAGAGC

TTCATGACCAAACCTGCCCTCTTGCCAATGTATTCTGTGAATATTGCAACACAATGC

TCATCAGGCAGCAGATGCCTAACCATTATGATAATGATTGTCCTACTGCCCCAGTAC

CATGCTTTTACAGTGCCTTTGGATGTCCTGAAAAGATGCAGAGGAATGAACTGGCA

CGACACATGCAGGAATTCACTCAGGTTCACATGAGAATGATGGCTCAGAGCATTCA

GAATATCAGTGTTACTGCTACAAACCCTGTACCCTTTATCAATGGCCTACCATTTGA

GCCTGCCCTCTTCTCCCATGTGTCACGTGCCCCGTATAATTGTAATCCAGAAGTTGA

AAACTTTAAAGAAACTATTCAGCAATTGGAAGGTCGTCTGGTGAGACAAGATCAC

CAAATCAGAGAACTTATTGCAAAGATGGAGACGCAAAACACTCACATGGCAGAAC

TCAAACGTACTATCCGAGATTTGGAGGGAAAGATTACTGAAATGGAGGCACAGCA

ATGTAATGGTATTTACATCTGGAAGATCGAGAACTTCAGTGGACTTCAGAAAGCCC

AGGAAGAAGAGAGACCTGTTGTGATGCACAGTCCCGGCTTCTATACTGGAAAACC

TGGCTACAAGCTGTGTTTGCGCCTGCATATCCAGTTACCAAGTGCTCAGCGGTGTG

CTAATTTTATATCTCTGTTTGTCCACACGATGCAAGGAGAATATGACAGCCATCTGC

CTTGGCCTTTCCAAGGCACTATACGACTTTCTATTTTGGATCAATCGGAGGGCCCTG

AAAGGCAGAATCATGAAGAAGTAATGGAAGCCAAGCCAGAGTTACTGGCCTTCCA

GAGACCAACAATTCACCGCAATCCAAAAGGTTTTGGTTATGTGACTTTCATGCACC

TGCAAACTTTGAAGCAGAGAACCTTTGTAAAGGATGATACCCTTCTGGTGCGCTGT

GAAGTTCTAACGCGTCTGGACTTAAACAGCCTTCGCAGGGAAGGATTTCAAGCTC

GCAGTACTGATGGAGCTGCGTAA

Supplementary Figures:

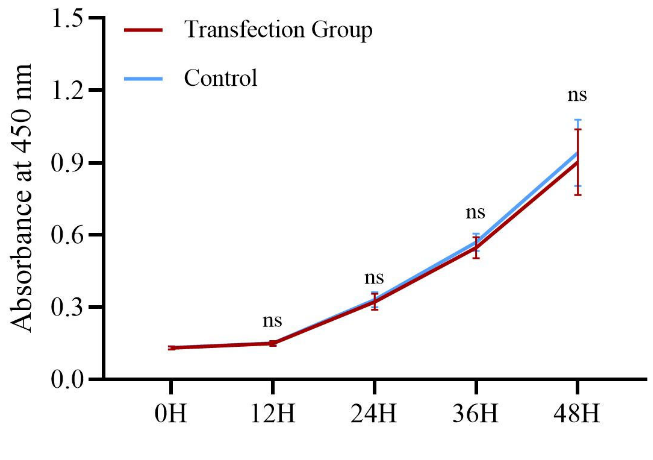

Figure S1. CCK-8 analysis was performed to evaluate the cell viability after the transfections. All data were presented as mean ± standard deviation, and at least six biological replicates per group. ^{ns} $P > 0.05$.

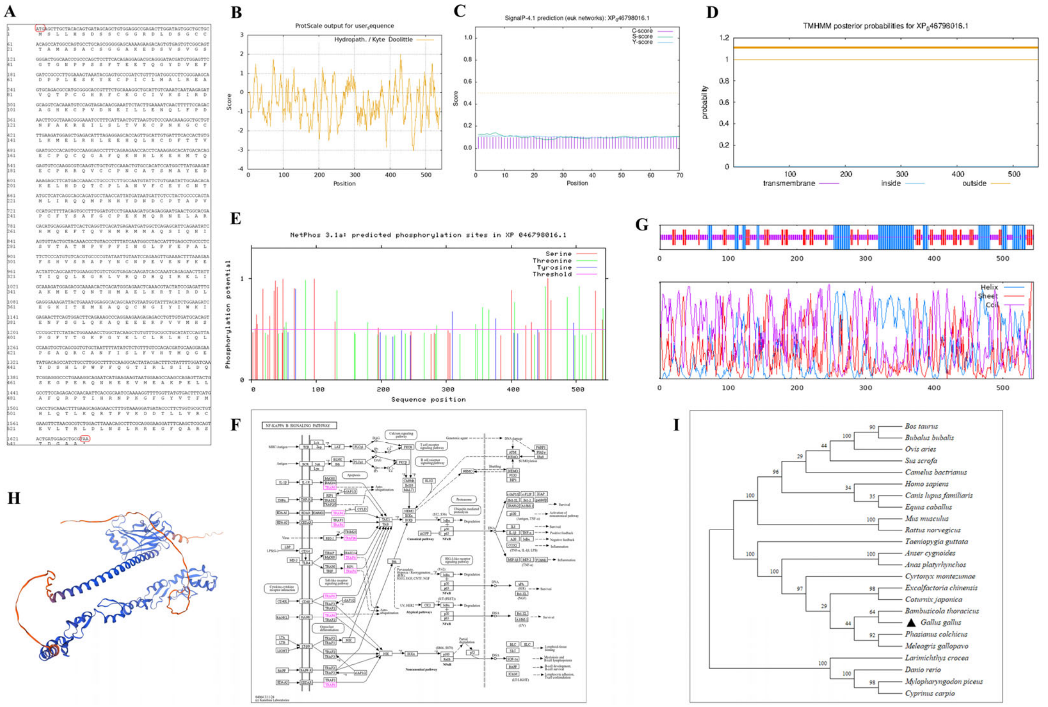

Figure S2. Bioinformatics characterization of chicken TRAF6. (A) Nucleotide sequence of the full-length coding sequence (CDS) and corresponding amino acid sequence of chicken TRAF6. (B) Hydrophilicity/hydrophobicity prediction of chicken TRAF6 protein. (C) Signal peptide prediction of chicken TRAF6 protein. (D) Transmembrane structure prediction of chicken TRAF6 protein. (E, F) predictions of phosphorylation sites and regulatory pathways of chicken TRAF6 protein. (G, H) Secondary and tertiary structure prediction of chicken TRAF6 protein. (I) Phylogenetic tree analysis of TRAF6 amino acid sequences among different species.

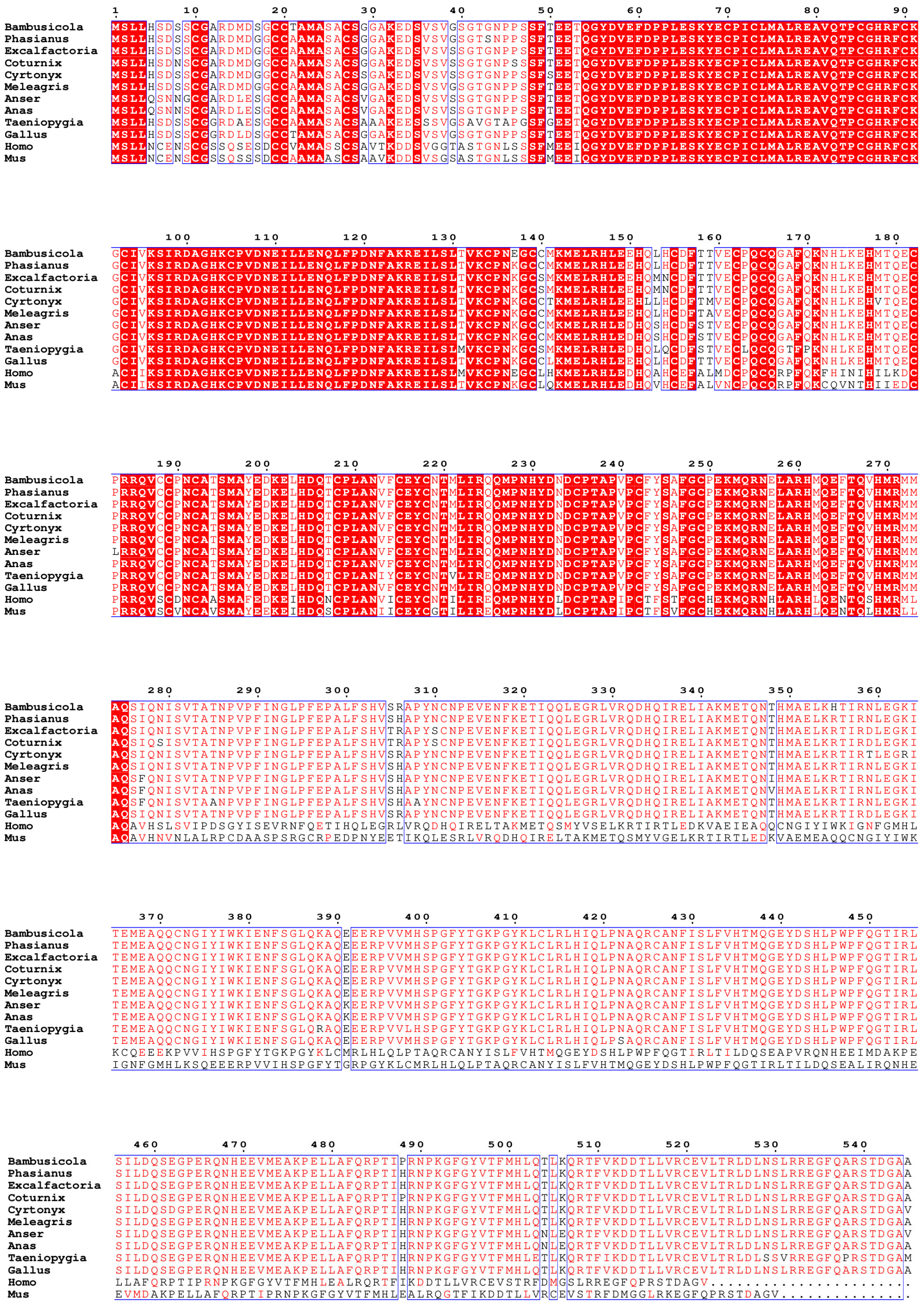

Figure S3. Multiple alignment analysis of TRAF6 amino acid sequence in different species.

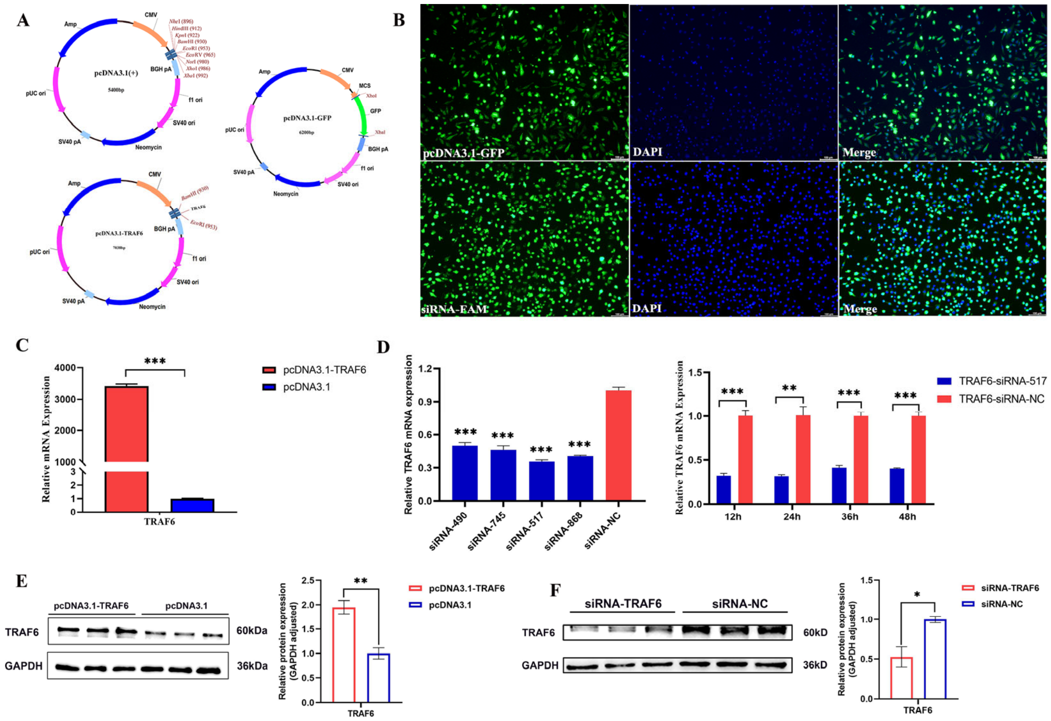

Figure S4. Validation of *TRAF6* overexpression and knockdown efficiency. (A) Schematic representation of the *TRAF6* overexpression recombinant vector pcDNA3.1. (B) Transfection efficiency assessment of pcDNA3.1-GFP plasmid and FAM-labeled siRNA oligonucleotide in DF-1 cells at 24 h post-transfection. DAPI was used to visualize cell nuclei. (C-D) Detection of *TRAF6* mRNA overexpression and knockdown efficiency in chicken DF-1 cells at 24 h post-transfection (n=3). (E-F) The evaluation of TRAF6 protein overexpression and knockdown efficiency in chicken DF-1 cells at 24 h post-transfection (n=3). *$P < 0.05$, **$P < 0.01$, ***$P < 0.001$.

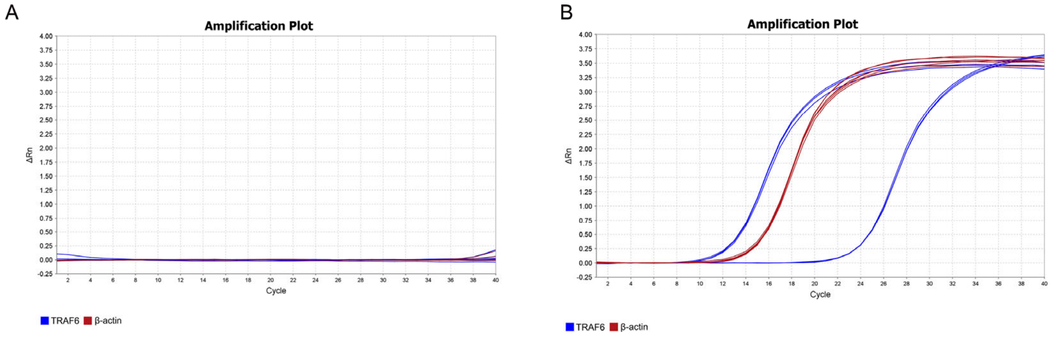

Figure S5. No-RT control validation for *TRAF6* overexpression.

(A) No-RT control; (B) Positive control (cDNA)

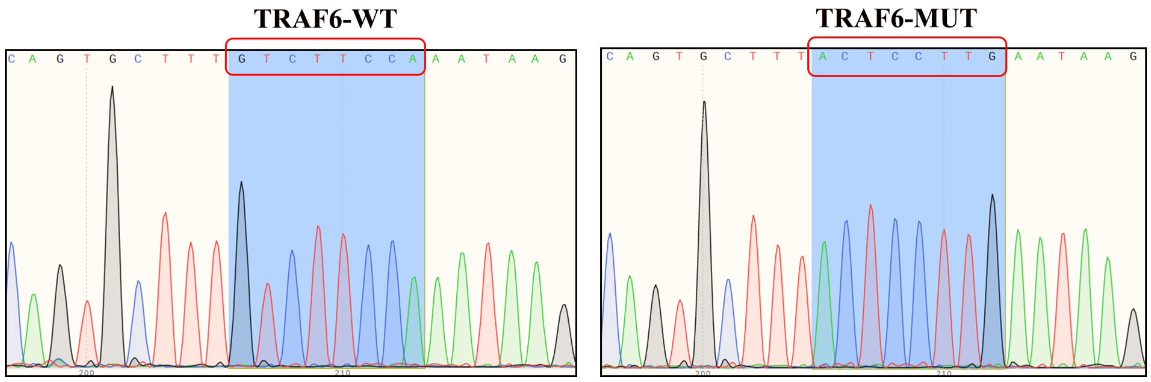

Figure S6. Sanger sequencing chromatograms of wild-type and mutant *TRAF6* 3′UTR recombinant plasmids.

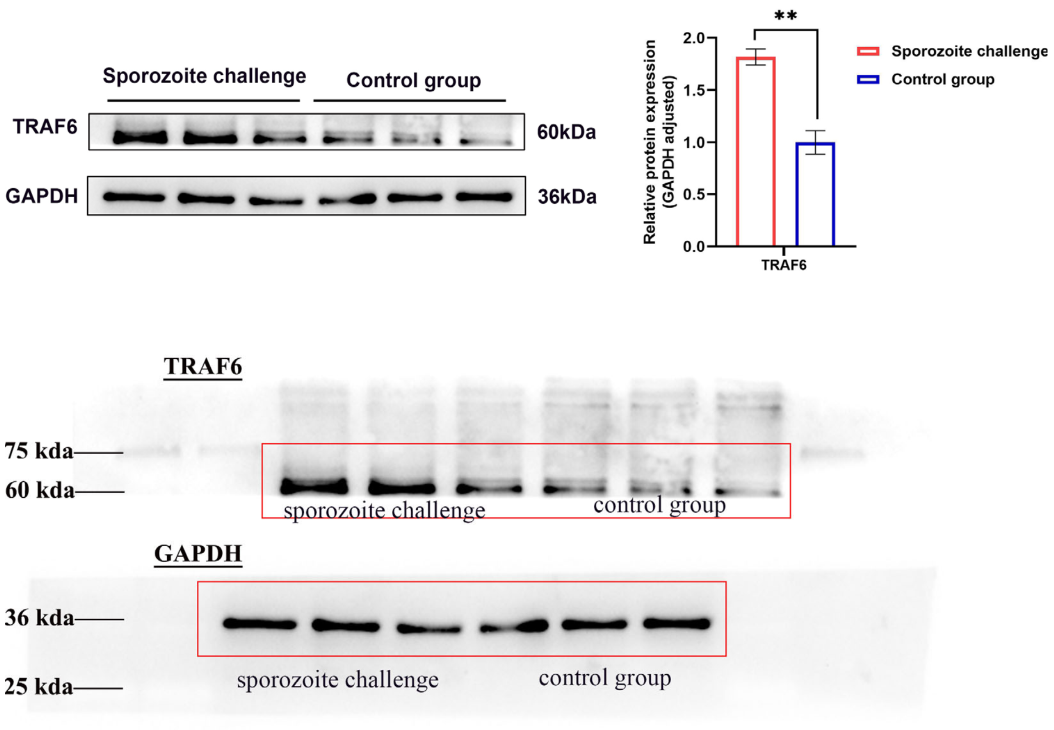

Figure S7. Original western blot images corresponding to Figure 3 in the main text, showing the protein levels of TRAF6 and GAPDH in *E. tenella* sporozoite-infected DF-1 cells. The membrane was cut with a scalpel to allow for simultaneous detection of TRAF6 and GAPDH on the same blot. The bands used in Figure 3 are marked with solid red boxes.

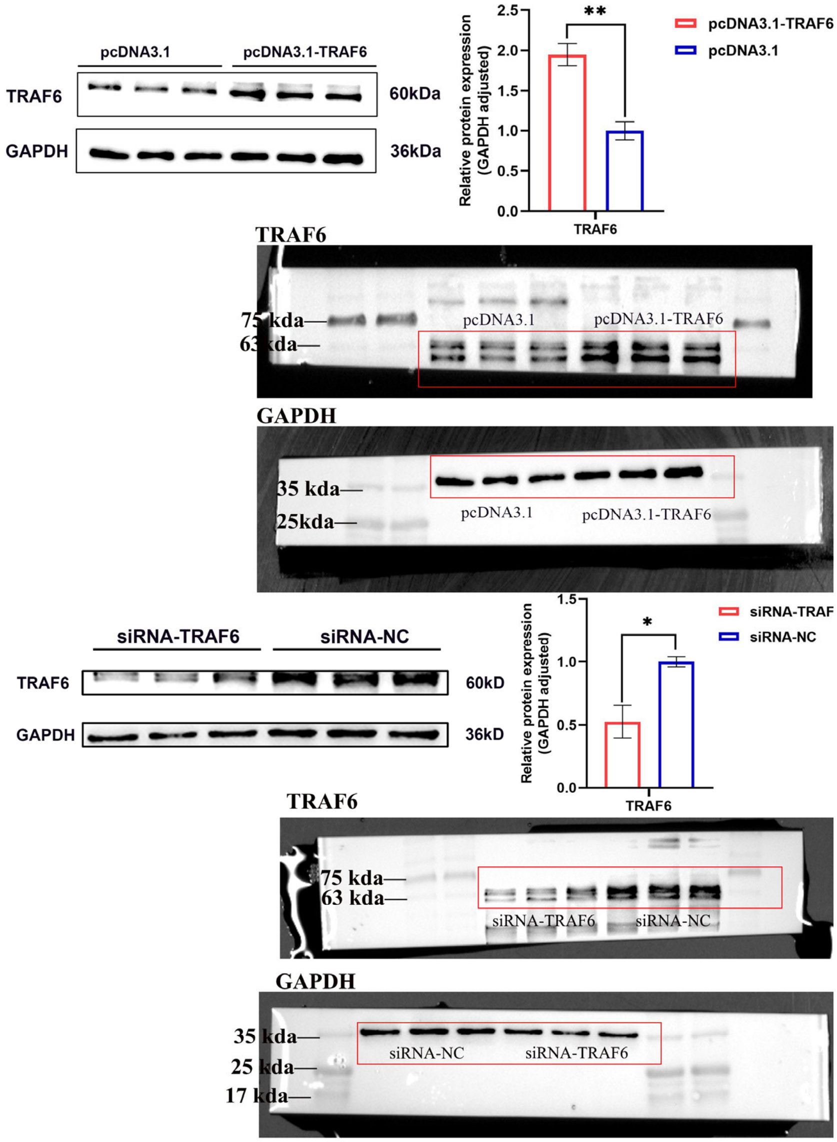

Figure S8. Original western blot images corresponding to Figure S4 in the main text, showing the protein levels of TRAF6 and GAPDH in DF-1 cells transfected with *TRAF6* overexpression and knockdown vectors. The membrane was cut with a scalpel to allow for simultaneous detection of TRAF6 and GAPDH on the same blot. The bands used in Figure S4 are marked with solid red boxes.

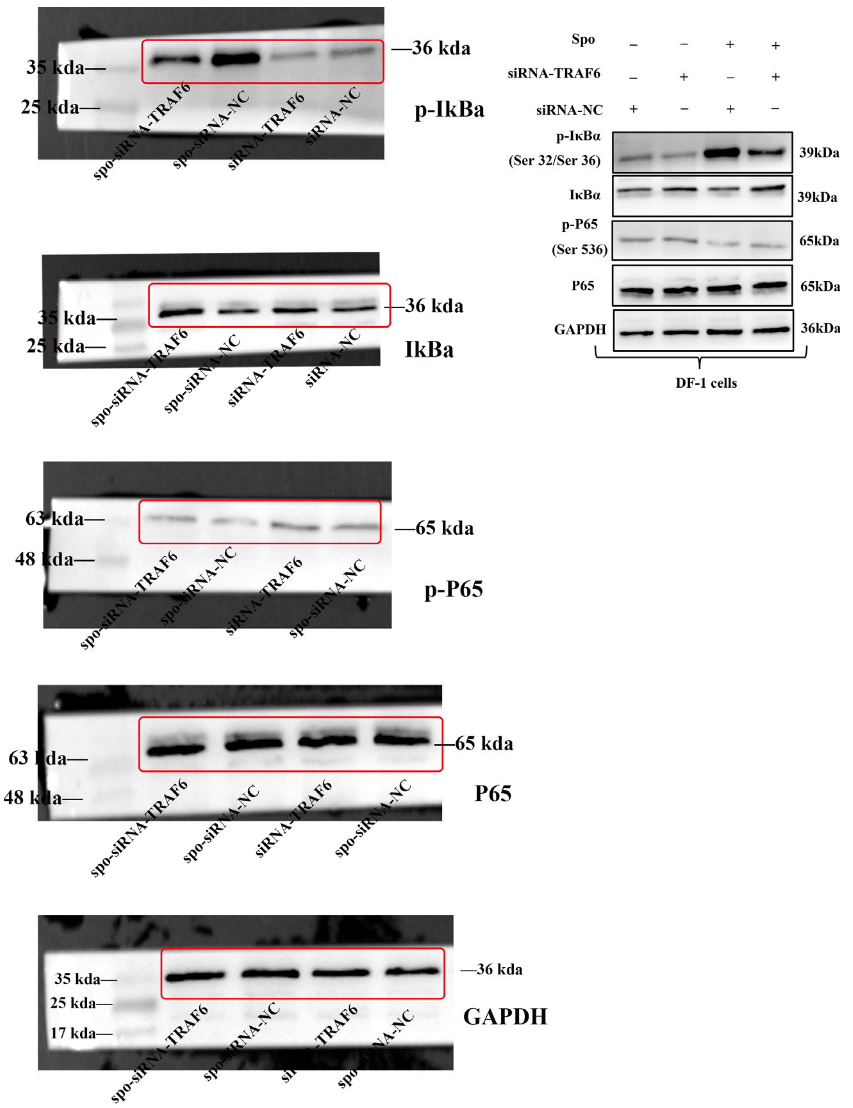

Figure S9. Original western blot images corresponding to Figure 4 in the main text, showing the protein levels of p-IκBα, IκBα, p-P65, P65, and GAPDH following *TRAF6* knockdown in DF-1 cells. The membrane was cut with a scalpel to allow for simultaneous detection of p-IκBα, IκBα, p-P65, P65, and GAPDH on the same blot. The bands used in Figure 4 are marked with solid red boxes.

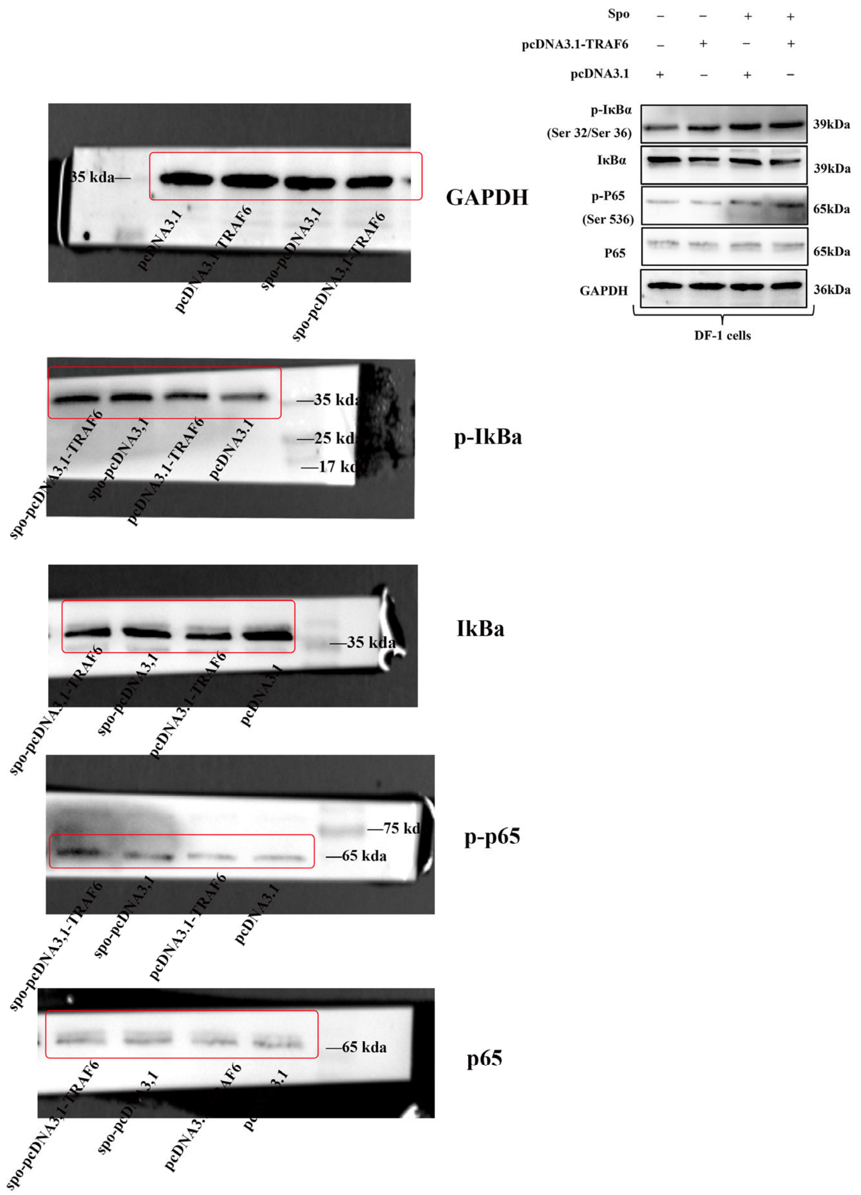

Figure S10. Original western blot images corresponding to Figure 5 in the main text, showing the protein levels of p-IκBα, IκBα, p-P65, P65, and GAPDH following *TRAF6* overexpression in DF-1 cells. The membrane was cut with a scalpel to allow for simultaneous detection of p-IκBα, IκBα, p-P65, P65, and GAPDH on the same blot. The bands used in Figure 5 are marked with solid red boxes.

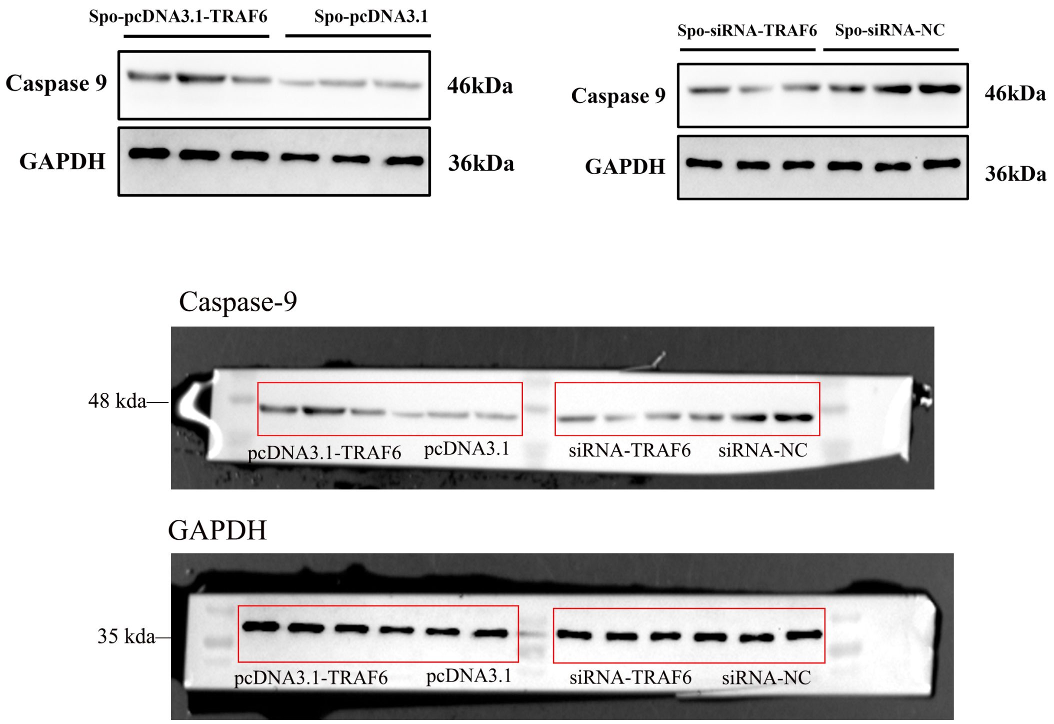

Figure S11. Original western blot images corresponding to Figure 6 in the main text, showing the protein levels of Caspase-9, and GAPDH in sporozoite-infected DF-1 cells transfected with *TRAF6* overexpression or interference and its control. The membrane was cut with a scalpel to allow for simultaneous detection of Caspase-9, and GAPDH on the same blot. The bands used in Figure 6 are marked with solid red boxes.

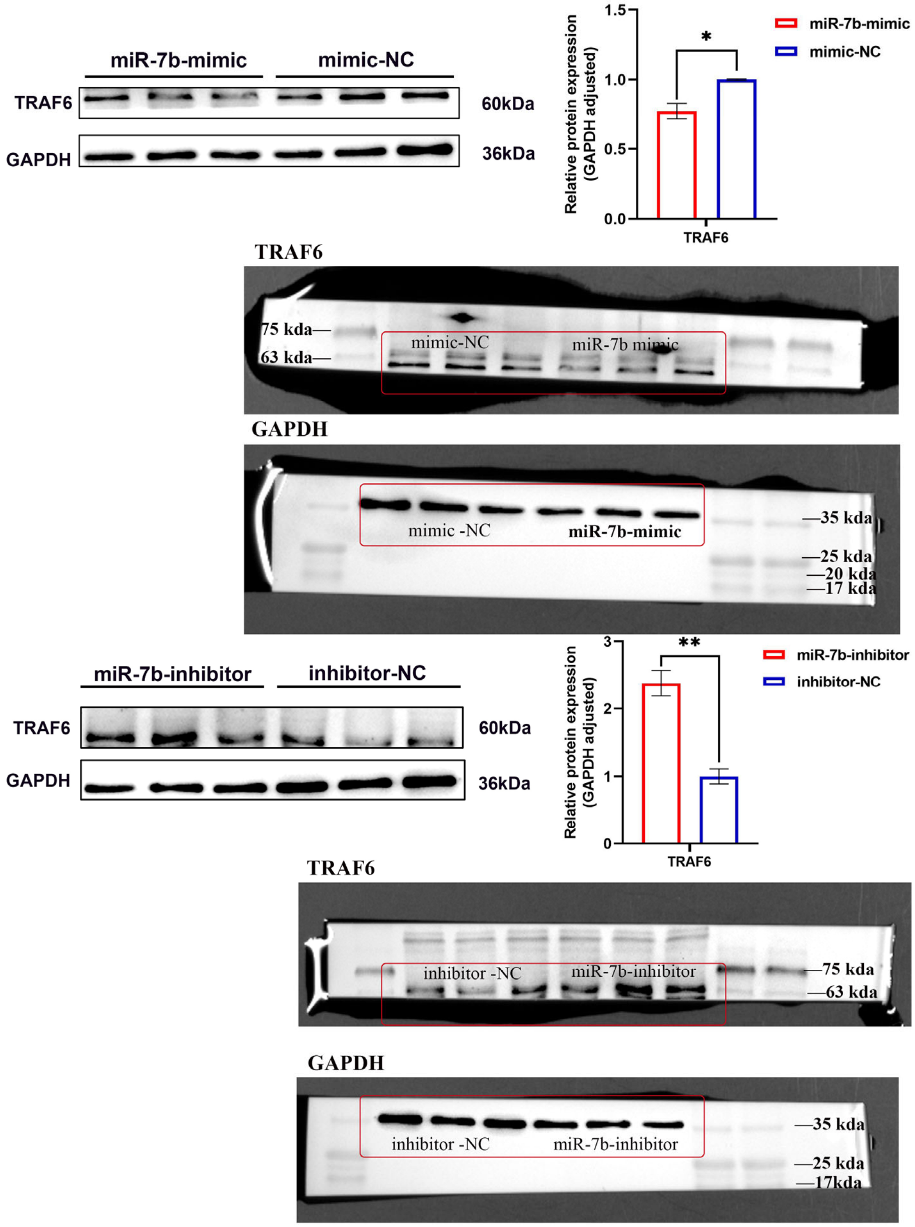

Figure S12. Original western blot images corresponding to Figure 7 in the main text, showing the protein levels of TRAF6, and GAPDH after in vitro transfection with gga-miR-7b mimic and its control, as well as gga-miR-7b inhibitor and its control. The membrane was cut with a scalpel to allow for simultaneous detection of TRAF6, and GAPDH on the same blot. The bands used in Figure 7 are marked with solid red boxes.

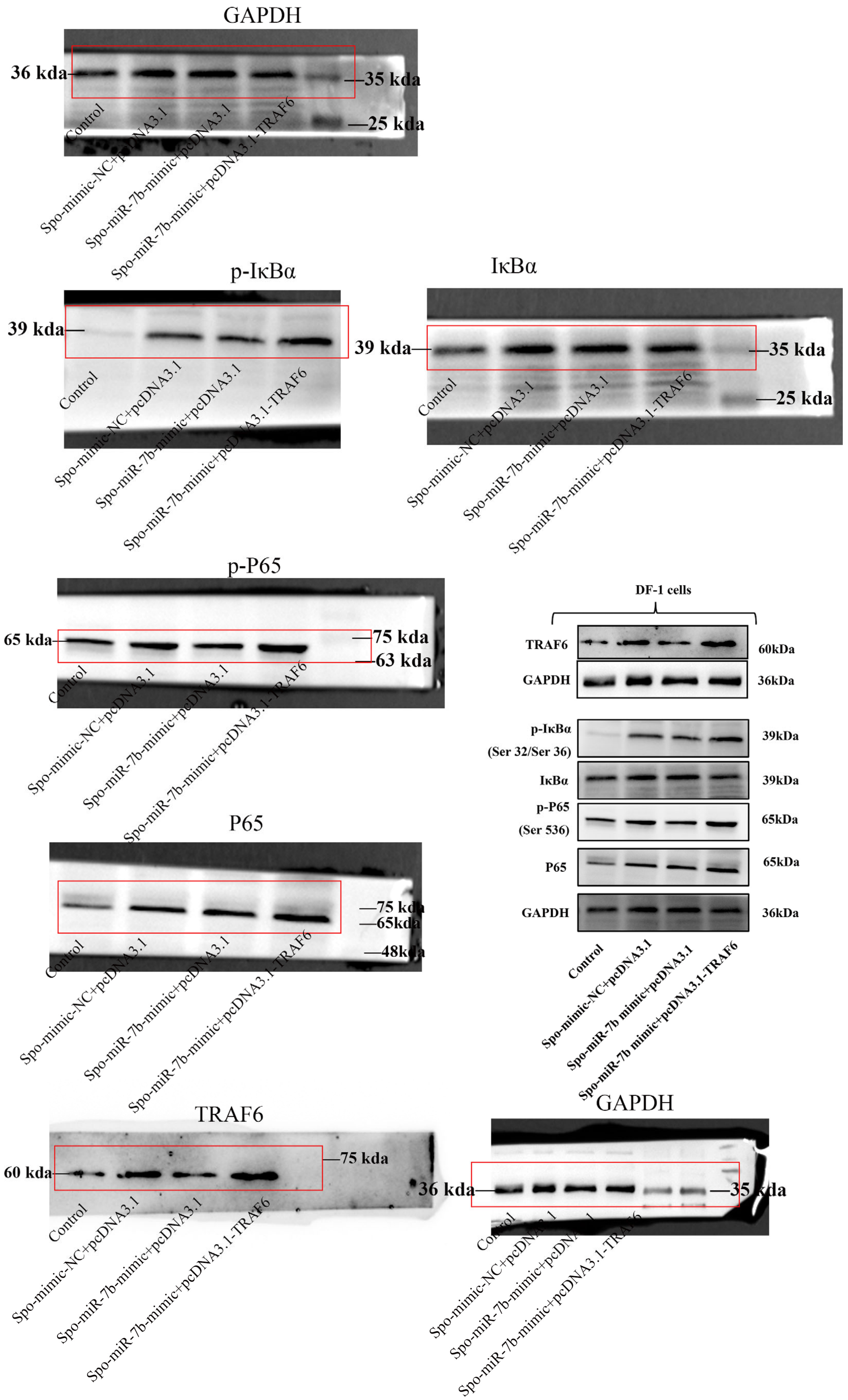

GAPDH
36 kda
35 kda
25 kda
Control
Spo-mimic-NC+pcDNA3.1
Spo-miR-7b-mimic+pcDNA3.1
Spo-miR-7b-mimic+pcDNA3.1-TRAF6
p-IκBα
39 kda
IκBα
39 kda
35 kda
25 kda
p-P65
65 kda
75 kda
63 kda
P65
75 kda
65kda
48kda
DF-1 cells
TRAF6
60kDa
GAPDH
36kDa
p-IκBα
(Ser 32/Ser 36)
39kDa
IκBα
39kDa
p-P65
(Ser 536)
65kDa
P65
65kDa
GAPDH
36kDa
Control
Spo-mimic-NC+pcDNA3.1
Spo-miR-7b mimic+pcDNA3.1
Spo-miR-7b mimic+pcDNA3.1-TRAF6
TRAF6
60 kda
75 kda
GAPDH
36 kda
35 kda

Figure S13. Original western blot images corresponding to Figure 8 in the main text, showing the protein levels of TRAF6, GAPDH, p-IκBα, IκBα, p-P65, and P65 after in vitro co-transfection with gga-miR-7b mimic and pcDNA3.1-TRAF6. The membrane was cut with a scalpel to allow for simultaneous detection of TRAF6, GAPDH, p-IκBα, IκBα, p-P65, and P65 on the same blot. The bands used in Figure 8 are marked with solid red boxes.
